# Disentangling sensory precision and prior expectation of change in autism during tactile discrimination

**DOI:** 10.1038/s41539-023-00207-5

**Published:** 2023-12-06

**Authors:** Laurie-Anne Sapey-Triomphe, Gaëtan Sanchez, Marie-Anne Hénaff, Sandrine Sonié, Christina Schmitz, Jérémie Mattout

**Affiliations:** 1 Université Claude Bernard Lyon 1, CNRS UMR5292, INSERM U1028, Centre de Recherche en Neurosciences de Lyon CRNL U1028 UMR5292, COPHY, F-69500 Bron, France; 2 Centre de Ressource Autisme Rhône-Alpes, Centre Hospitalier Le Vinatier, Bron, France; 3 Hôpital Saint-Jean-de-Dieu, Lyon, France

**Keywords:** Perception, Autism spectrum disorders

## Abstract

Predictive coding theories suggest that core symptoms in autism spectrum disorders (ASD) may stem from atypical mechanisms of perceptual inference (i.e., inferring the hidden causes of sensations). Specifically, there would be an imbalance in the precision or weight ascribed to sensory inputs relative to prior expectations. Using three tactile behavioral tasks and computational modeling, we specifically targeted the implicit dynamics of sensory adaptation and perceptual learning in ASD. Participants were neurotypical and autistic adults without intellectual disability. In Experiment I, tactile detection thresholds and adaptation effects were measured to assess sensory precision. Experiments II and III relied on two-alternative forced choice tasks designed to elicit a time-order effect, where prior knowledge biases perceptual decisions. Our results suggest a subtler explanation than a simple imbalance in the prior/sensory weights, having to do with the dynamic nature of perception, that is the adjustment of precision weights to context. Compared to neurotypicals, autistic adults showed no difference in average performance and sensory sensitivity. Both groups managed to implicitly learn and adjust a prior that biased their perception. However, depending on the context, autistic participants showed no, normal or slower adaptation, a phenomenon that computational modeling of trial-to-trial responses helped us to associate with a higher expectation for sameness in ASD, and to dissociate from another observed robust difference in terms of response bias. These results point to atypical perceptual learning rather than altered perceptual inference per se, calling for further empirical and computational studies to refine the current predictive coding theories of ASD.

## Introduction

Autism Spectrum Disorders (ASD) refer to a range of highly prevalent neurodevelopmental disorders, characterized by heterogeneous behavioral symptoms: difficulties in social interactions and communication, repetitive behaviors, and restricted interests (DSM V)^1^. The DSM V also highlights the importance of perceptual symptoms in ASD, such as the hyper responsiveness to sensory stimuli^1^. While some of the first theories focused on the social symptoms^2^, others have addressed the atypical perception in ASD defined by a decreased global processing and increased local processing^3,4^. Theories, cast in a computational (Bayesian) framework, propose more generic mechanisms in an attempt to explain both social and non-social symptoms in ASD^5–10^. An ongoing and thought-provoking theoretical debate aims at accounting for the symptoms encountered in ASD, as the expression of atypical probabilistic inference and learning^6,8–13^. In particular, an atypical predictive coding system would have consequences on their social life, as inferring others’ intentions and their evolutions is crucial^14,15^. Refining the predictive coding theories of ASD could thus help highlighting the core mechanisms at play^16,17^. Grounded on empirical evidence, the present study aims to contribute to this endeavor to shed light on the mechanisms underlying perceptual inference and learning in ASD.

In Bayesian terms, sensory information is combined with prior beliefs to generate percepts (*posteriors*). The relative contributions (or weights) of prior and sensory evidence reflect their respective precision or confidence. Importantly, these precisions also have to be inferred from sensory inputs. The hypothesis of a different Bayesian inference has been put forward in ASD, namely, an atypical balance between prior and sensory precisions could account for the symptoms encountered in ASD^6,8–10,12,13^. At least three mechanistic hypotheses of atypical weighting of priors and sensory inputs in ASD have been formulated. The *hypo-prior* hypothesis^10^ suggests that priors are blurred in ASD, so that they do not influence perception. This is consistent with the idea that autistic people tend to be less influenced by priors in optical illusions^18,19^. In contrast, the high sensory accuracy hypothesis suggests that sensory information could be afforded a very high precision when computing the percept^9,12^. The idea of a high sensory precision in ASD is in line with their hypersensitivity and acute discrimination abilities^20,21^. Note that this second hypothesis appears to be non-trivially distinguishable from the first one, simply because the sensory precision and prior precision play a symmetric role in Bayesian inference^9^. As a consequence, they can only be disentangled through their differential effect on second order statistics (posterior precisions)^11^. Finally, a third hypothesis points to a High and Inflexible Precision of Prediction Error in Autism (HIPPEA)^6^. Interestingly, this hypothesis refers to the precision weighting of prediction errors, which involves the posterior precision, i.e., both the sensory and prior precisions^22^. More importantly, it does not only invoke the precision values or estimates, but also their adjustment over time. This is important as it not only speaks to the influence of precision on local (trial wise) perceptual inference, but to the process of adjusting the relative confidence of sensory and prior information through perceptual learning (over trials and context). Typically, a difficulty in attributing accurate precisions would give rise to context-insensitive prediction errors and would systematically challenge the existing priors, because noisy sensory inputs would be erroneously considered as relevant. High sensory precision at the early stages of sensory processing would lead to upregulated prediction errors, which could induce narrow priors hardly adapted in changing contexts. This emphasizes the importance of the relative sensory and prior precisions, and of the dynamics in the adjustment of those precisions^6,8^.

Such theories point to core mechanisms in ASD that would explain both non-social and social symptoms. Importantly, they also lay the ground for quantitative computational predictions that can be tested empirically. Identifying such a mechanism could contribute to improving diagnoses of ASD and opening new avenues for interventions. Assessing and quantifying precision-weighted priors and sensory inputs is challenging because these quantities are updated in an inter-dependent fashion. This challenge is acute, which explains the relative scarcity of current evidence in favor of a precise computational account of ASD. Studies investigating prior influence on perception suggested intact priors in individuals with high autistic traits^23^ and with ASD^24–29^ or reduced prior influence in autistic individuals^30,31^ (for reviews, see refs. ^32,33^). Indeed, a recent review^33^ concluded that the majority of the studies testing the predictive coding theories of ASD did not support a prior-likelihood imbalance in ASD, even though a third of the studies pointed toward a reduced prior weight in ASD. In particular, paradigms using implicitly learned priors were more likely to reveal a prior/-likelihood imbalance, in contrast with paradigms using explicitly learned or pre-existing priors^33^. The authors highlighted the need to include computational approaches to better investigate these predictive coding theories in ASD^33^. Furthermore, learning mechanisms have been often reported as atypical in ASD, which could affect the way priors are built up. Indeed, autistic individuals show more difficulties extracting regular patterns that underlie their experience of the world^34,35^, or have been characterized by a too-specific learning^36,37^, a slower learning^38^, an atypical learning in a volatile environment^36,39^ but also a typical learning^40^ (see^32^ for a review). Interestingly, among the few approaches using computational models, a recent study suggested a difference in the dynamics of perceptual inference, as autistic individuals weighted recent stimuli less heavily to update their priors, as compared to neurotypicals^38^. Studies using an electrophysiological or neuroimaging approach suggested more inflexible prediction errors in ASD^41^, and (sometimes atypically but) hierarchically organized predictions and prediction errors in ASD^42^. Finally, there is evidence for reduced (but also sometimes intact) sensory adaptation in ASD^43–49^. Here, we refer to sensory adaptation as a process which reduces redundancy by biasing perception away from repeated features in the environment, making novel stimuli and features more salient^43^.

**Fig. 1:**
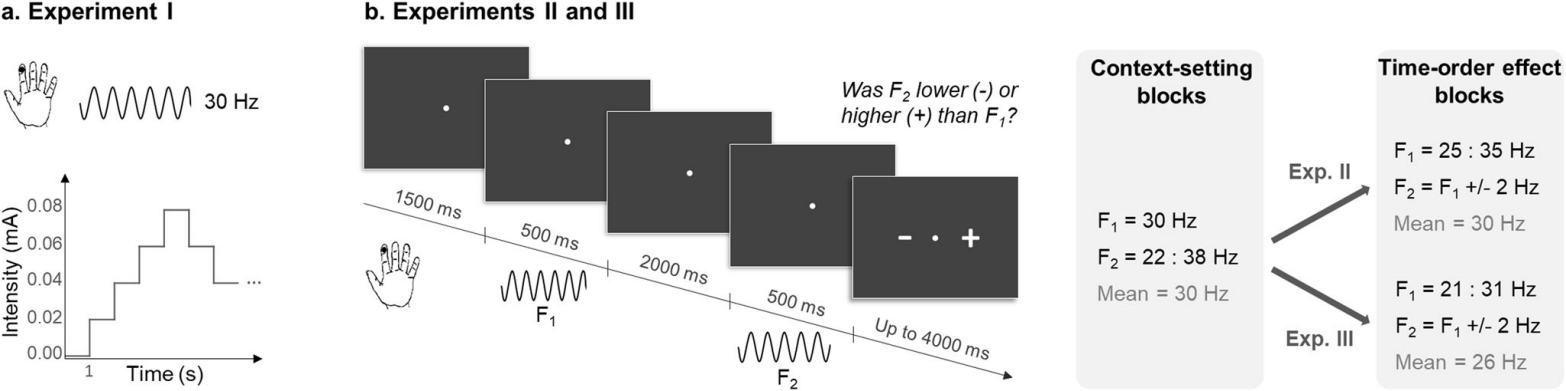
Experimental paradigms. **a** Tactile detection threshold measurement: while the frequency was set at 30 Hz, the intensity of the stimulation gradually increased or decreased (methods of limit) to determine the participant’s threshold. **b** Two-alternative forced-choice task: participants had to determine whether the frequency of the second stimulation (*F*_2_) was higher or lower than the first one (*F*_1_). The frequencies used for Experiments II and III are detailed in the right part of the Figure (3 context-setting blocks centered on 30 Hz, followed by two time-order effect blocks centered on 26 or 30 Hz).

For significant progress to be made, one central need is a task that would both elicit a behavioral effect in autistic participants and enable the precise identification of a mechanistic account of this effect. In such an attempt, we use a simple and low-level tactile frequency discrimination task designed to elicit a time-order effect (TOE) (Fig. 1b). This robust and well-documented effect relates to the contraction bias described in the Central tendency of judgment^50–59^, similar to the effect of regression towards the mean^60,61^. A contraction bias emerges in sequential judgments of stimuli along a particular dimension (e.g., frequency), where stimulations seem closer to the prior (e.g., mean frequency) than they really are. It leads to the overestimation of stimuli lying in the lower range of stimulations, and to the underestimation of those lying in the upper range. The TOE is subsequent to this bias and can be observed in two-alternative forced choice tasks (2AFC), where the strength of the contraction bias differs between the two stimulations. It occurs as if the percept of the first stimulus would be more biased towards the prior, which some authors have related to the need for that stimulus to be stored in memory during the delay period^56^. This effect is independent of the sensory modality and can be massive^53^. It is thought to reflect the influence of acquired *priors* built on the recent history of perceived stimulations during the task^62^. Importantly, this is in the case of uncertain or ambiguous environments, hence difficult decisions, that the TOE emerges and that the most pervasive difficulties of ASD should be observed^12^. TOE tasks can highlight the processes of belief updating (learning) and relative precision tuning in a context-dependent fashion (meta-learning).

Following the HIPPEA hypothesis^6^, several subtle predictions can be made on prior construction and sensory adaptation in ASD in our perceptual task. First, we assume that a long-lasting exposure to a stable experimental context would enable autistic participants to build up a precise and strongly influencing prior. Indeed, having a high and inflexible precision ratio would lead to a more precise prior only in the specific context where stimuli would never vary (i.e., like in the context-setting blocks of our task). Note that in a more changing or variable context (i.e., more naturalistic situation), the prior precision would be lower in ASD, in line with the weak prior hypothesis^10^. Hence, in our task, the TOE should not only be visible, but even stronger in autistic participants compared to neurotypical participants. Second, in a varying environment, their more inflexible precision weighting would reveal a maladaptive behavior. In other words, when suddenly changing the frequency range, the TOE should be adjusted to the new range slower in autistic than neurotypical participants. Another consequence of a more inflexible precision weighting would be a decreased sensory adaptation in the ASD group, as it reflects the flexible suppression of prediction errors for predictable stimuli. Finally, we assume that the precision ratio would be less flexibly modulated in ASD following changes in the environmental structure.

We tested these predictions with three experiments in NT and ASD adults, and used a computational model of 2AFC to further emphasize the relationship between weighted prediction errors and behavioral responses. Using a dynamic perceptual decision-making model (assuming learning over trials) and a static one (assuming no learning), we assessed the adjustment of the sensory/prior precision ratio in response to contextual changes. The aim is to contribute to elucidating whether the predictive coding hypotheses of ASD (e.g., weak priors^10^, high sensory precision^9^ or HIPPEA^6^) suffice to characterize ASD functioning or need to be refined.

## Results

### Absolute detection thresholds and sensory adaptation (Exp. I)

In Exp. I, the average intensity detection threshold of 30 Hz stimulations did not differ between the neurotypical (NT) group (0.6 mA ± 0.2) and the ASD group (0.6 mA ± 0.4) (*t*(61) = 0.3, *p* = 0.79, *d* = 0.68). There was moderate evidence in favor of an absence of group difference (BF = 0.27). Note that in each discrimination experiment, absolute detection thresholds did not differ between groups either (Exp. II: 0.5 mA ± 0.2 in both groups, *t*(31) = 0.2, *p* = 0.84, *d* = 0.07, BF = 0.34; Exp. III: 0.6 mA ± 0.2 in NT, 0.5 mA ± 0.2 in ASD, *t*(35) = 0.9, *p* = 0.36, *d* = 0.07, BF = 0.44). Therefore, NT and ASD adults did not differ in mean tactile detection thresholds.

**Fig. 2:**
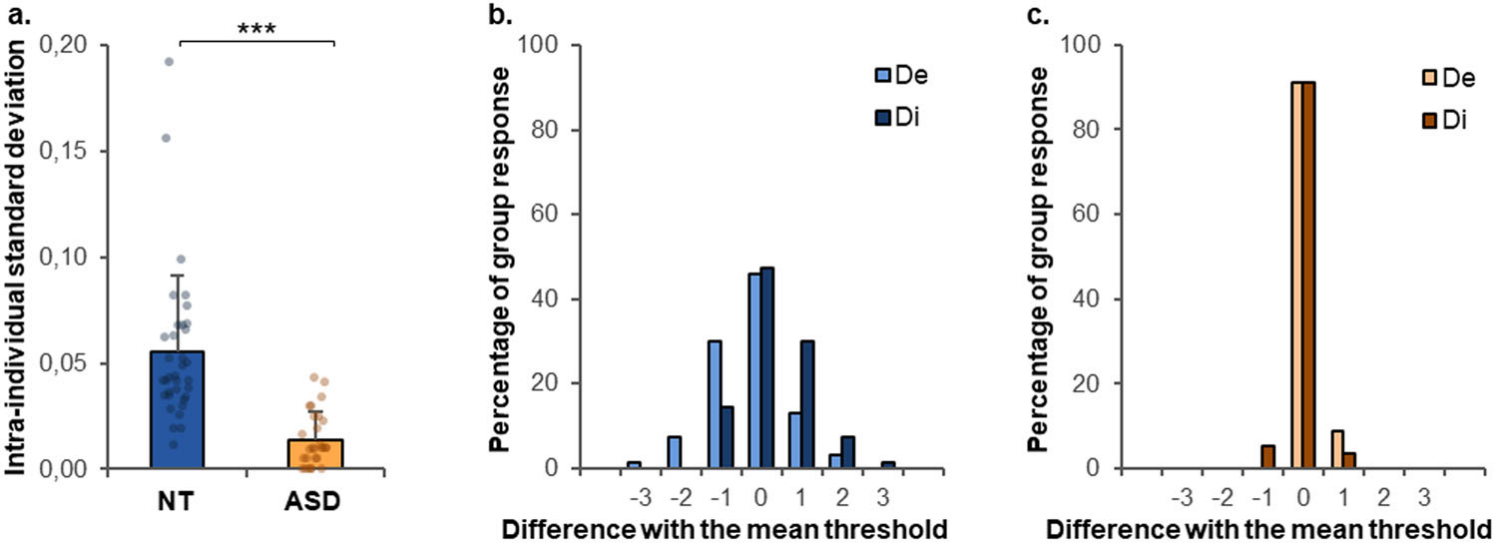
Tactile detection threshold and sensory adaptation in Experiment I. **a** Intra-individual standard deviation of the detection threshold measurements in neurotypical (NT) and autistic (ASD) participants. Error bars represent the standard deviations. ***p < 0.0001. **b**, **c** Cumulated distribution of the individual dispersion of the perception threshold across participants in the NT (**b**) and ASD (**c**) groups. The *x*-axis presents the percentage of measures which differed by x mA from the mean threshold of the participant: unit −3: ≤ −0.15 mA, unit -2: [−0.15 to −0.09[, unit −1: [−0.09 to −0.03[, unit 0: [−0.03 to 0.03[, unit 1: [0.03 to 0.09[, unit 2: [0.09 to 0.15[, unit 3: ≥0.15 mA. De/Di detection/disappearance of the stimuli.

Rather than only measuring the average of the absolute detection threshold, we also assessed how variable or consistent the detection threshold measures were within each participant, as a high sensory precision should lead to low intra-individual variability. The intra-individual standard deviation of the four detection threshold measures was much higher in the NT group (0.06 ± 0.04) than in the ASD group (0.01 ± 0.01) (*t*(61) = 5.8, *p* < 0.0001, *d* = 1.46; Fig. 2a). There was extreme evidence in favor of a group difference (BF = 4.08 × 10^4^).

Furthermore, there was a sensory adaptation effect in the NT group (0.08 ± 0.16, *t*(34) = 2.9, *p* < 0.01, *d* = 0.49; Fig. 2b), but not in the ASD group (−0.01 ± 0.04, *t*(27) = 0.9, *p* = 0.38, *d* = 0.17; Fig. 2c). There was substantial evidence in favor of both adaptation in the NT group (BF = 6.39), and no adaptation in the ASD group (BF = 0.29). This sensory adaptation effect was significantly higher in NT than ASD participants (*t*(61) = 2.8, *p* < 0.01, *d* = 0.70). There was substantial evidence in favor of a group difference (BF = 5.85).

In sum, autistic adults were more precise than NT, that is, autistic participants showed very few variations in detection thresholds across several measurements. In contrast, NT participants showed more variability but, interestingly, this variability appears to be structured and reveals an adaptation effect that is not observed in ASD participants. Sensory adaptation, here, reflects the flexible suppression of errors for predictable stimuli.

### Behavioral results in the context-setting blocks (Exp. II and III)

**Fig. 3:**
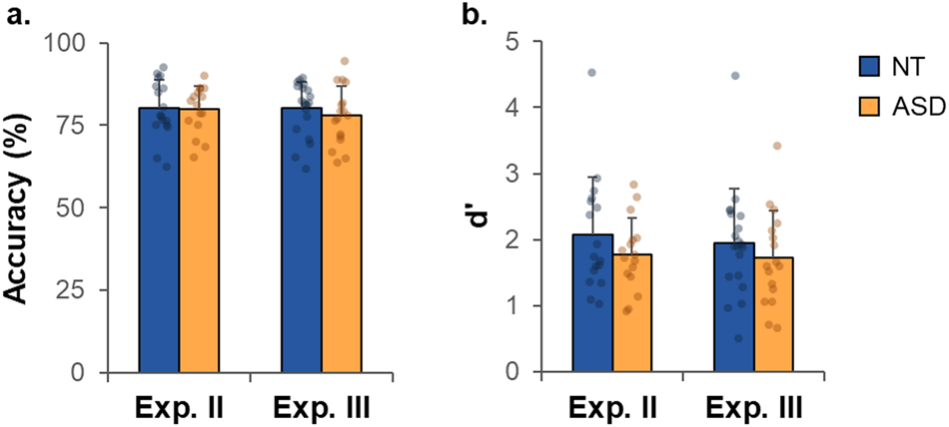
Sensory precision in the context-setting blocks of Experiments II and III. **a** Mean accuracy in the context-setting blocks (average over the three blocks). **b** D prime in the context-setting blocks (average over the three blocks). Error bars correspond to the standard deviations. Exp. experiment, NT neurotypical, ASD autism spectrum disorder.

In Exp. II, the percentages of correct answers were 81% (± 8) in NT and 79% (± 7) in ASD and did not differ between groups (*t*(31) = 0.8, *p* = 0.40, *d* = 0.29, BF = 0.43; Fig. 3a). There was no group difference in *d’* (*t*(31) = 1.2, *p* = 0.25, *d* = 0.40, BF = 0.56, Fig. 3b). The average response times did not differ between groups either (NT: 930 ms ± 432, ASD: 737 ms ± 252, *t*(31) = 1.6, *p* = 0.13, *d* = 0.54, BF = 0.82). The relative thresholds are shown as Supplementary Fig. 1 (group-level fits: NT: 2.7 Hz, ASD: 3.3 Hz), and did not differ between groups (subject-level fits: NT: 3.3 ± 2.7 Hz, ASD: 3.6 ± 2.1 Hz, *t*(31) = 0.4, *p* = 0.71, *d* = 0.13, BF = 0.35). As performance did not differ between groups in these context-setting blocks, it suggests a similar sensory precision in NT and ASD.

In Exp. III, the percentages of correct answers were 81% (± 8) in the NT group and 78% (± 9) in the ASD group (Fig. 3a) (no group difference, *t*(35) = 0.9, *p* = 0.36, *d* = 0.31, BF = 0.45). There was no group difference in *d*′ (*t*(35) = 0.9, *p* = 0.37, *d* = 0.30, BF = 0.44, Fig. 3b). The average response times did not differ between groups (NT: 751 ms ± 315, ASD: 876 ms ± 369, *t*(35) = 1.1, *p* = 0.27, *d* = 0.37, BF = 0.52). The relative thresholds are shown in Supplementary Fig. 1 (group-level fits: NT: 3.0 Hz, ASD: 3.5 Hz). Relative thresholds did not differ between groups (subject-level fits: NT: 3.6 ± 3.0 Hz, ASD: 4.5 ± 3.5 Hz, *t*(35) = 0.9, *p* = 0.38, *d* = 0.30, BF = 0.44). These results showing no group difference in performance also suggest a typical tactile precision in ASD.

### Behavioral results in the TOE blocks with a stable stimulus frequency range (Exp. II)

In the TOE blocks of Exp. II, there was no difference in accuracy between groups (NT: 69% ± 8, ASD: 66% ± 5, *t*(31) = 1.5, *p* = 0.14, *d* = 0.53, BF = 0.80). Autistic participants answered faster than NT participants (629 ms ± 206 in ASD vs. 899 ms ± 449 in NT, *t*(31) = 2.2, *p* = 0.036, *d* = 0.76, BF = 1.98). Furthermore, autistic participants answered faster in TOE blocks than in context-setting ones (629 ms ± 206 vs. 737 ms ± 252, *t*(15) = 2.7, *p* = 0.017, *d* = 0.67, BF = 1.31), whereas no RT differences were found within the NT group (*t*(16) = 0.9, *p* = 0.39, *d* = 0.21, BF = 0.32).

**Fig. 4:**
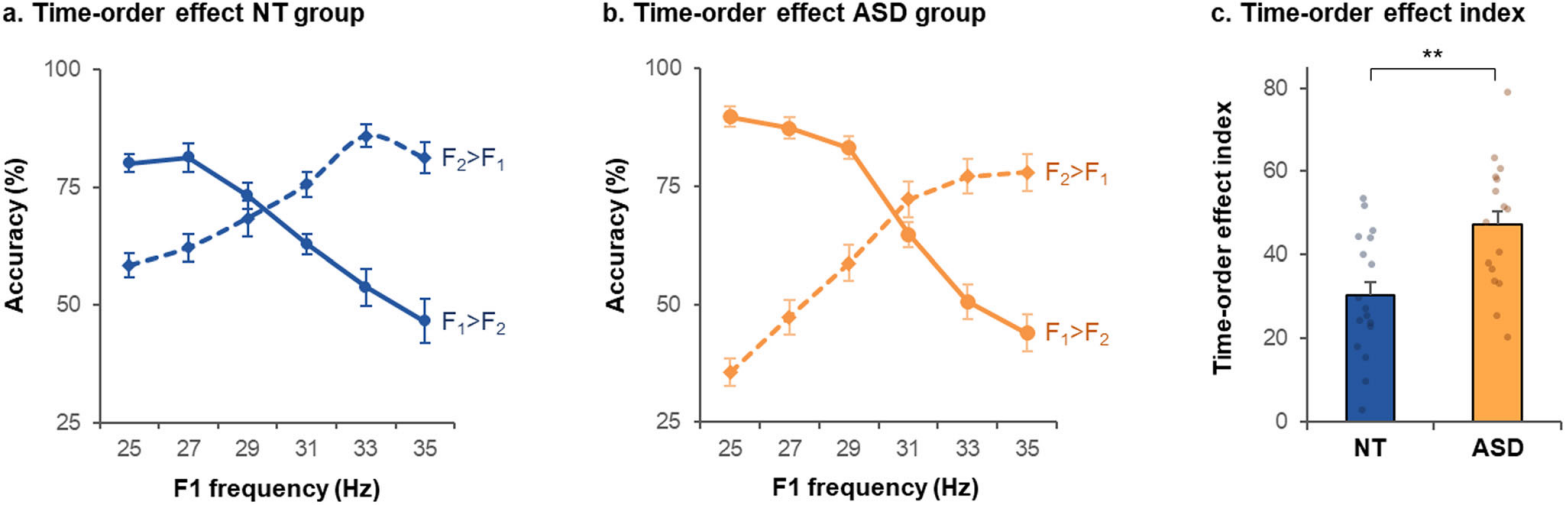
Time-order effect in Experiment II. **a**, **b** Percentage of correct answers in the TOE blocks in the neurotypical (NT) group (**a**) and in the autistic (ASD) group (**b**). “*F*_2_ > *F*_1_”: trials with *F*_2_ 2 Hz higher than *F*_1_, “*F*_2_ < *F*_1_”: trials with *F*_2_ 2 Hz lower than *F*_1_. **c** Time-order effect index in the TOE blocks. Error bars correspond to the standard error of the mean. **p < 0.005.

The index of TOE (*I*_TOE_) reflects how accuracy level varies depending on the stimulus pair presented at each trial and should be different from zero if perceptual decisions are influenced by the underlying statistics of the task. Both groups exhibited a TOE (Fig. 4a, b), with an *I*_TOE_ significantly different from zero in NT (*I*_TOE_ = 30 ± 15, *t*(16) = 8.5, *p* < 0.0001, *d* = 2.1, BF = 5.98 × 10^4^) and ASD (*I*_TOE_ = 47 ± 16, *t*(15) = 12.1, *p* < 0.0001, *d* = 3.0, BF = 2.88 ×10^6^).

As a high *I*_TOE_ reflects a strong influence of prior (relative to sensory inputs) on perceptual decisions, the *I*_TOE_ was compared between groups to assess if the relative weight of priors would be greater in one group. The *I*_TOE_ was significantly higher in ASD than in NT (*t*(31) = 3.2, *p* < 0.005, *d* = 1.1) (Fig. 4c). There was strong evidence in favor of a group difference (BF = 11.3). Importantly, the ASD group was highly homogeneous, as only two autistic participants scored slightly below the median *I*_TOE_ of the NT group (median: 27). The two-way nested ANOVA assessing the effect of group and TOE blocks (4 and 5) on the *I*_TOE_ only revealed a group effect (*F*(1,31) = 10.1, *p* < 0.01). As the TOE seems asymmetrical on Fig. 4, we conducted an ANOVA on the *I*_TOE_ assessing the effect of frequency (three *F*_1_ values lying below the mean vs. three *F*_1_ values above the mean) and group. There was a group effect (*F*(1,31) = 10.0, *p* < 0.005), with a higher *I*_TOE_ in ASD than NT. There was also an interaction between group and frequency (*F*(1,31) = 4.5, *p* = 0.041), with a significantly higher *I*_TOE_ in ASD than NT in lower frequencies (*t*(31) = 4.2, *p* < 0.001, *d* = 1.46, BF = 108) but not in higher frequencies (*t*(31) = 0.4, *p* = 0.68, *d* = 0.14, BF = 0.35).

As the TOE should be centered on the mean frequency of the stimulus range (i.e., 30 Hz), we assessed the intercept of the two accuracy curves (*F*_2_ > *F*_1_ and *F*_2_ < *F*_1_) in the TOE blocks. The intercept of the two accuracy curves (*F*_2_ > *F*_1_ and *F*_2_ < *F*_1_) did not differ between groups in any TOE block (first TOE block: *t*(27) = 1.1, *p* = 0.30, *d* = 0.61, BF = 0.53; second TOE block: *t*(28) = 1.5, *p* = 0.13, *d* = 0.56, BF = 0.84). In the NT group, the intercept was 29.6 Hz (± 3.7) in the first TOE block and 29.4 Hz (± 3.0) in the second TOE block. In the ASD group, the intercept was 30.9 Hz (± 2.7) in the first TOE block and 30.9 Hz (±2.3) in the second TOE block.

In sum, the presence of a TOE in both groups reveals that both NT and ASD participants implicitly learned a prior (i.e., about the frequency range of the delivered tactile stimulations) which biased their percepts, and therefore led to a modulation of their accuracy across trials, despite no objective change in task difficulty. As autistic participants showed a stronger TOE than NT, it indicates a stronger influence of the prior (relative to sensory inputs) on perception in ASD in Exp. II.

### Behavioral results in the TOE blocks after a change in stimulus frequency range (Exp. III)

In the TOE blocks of Exp. III, the average percentage of correct answers and response times were 67% (± 6) and 718 ms (± 318) in the NT group and 65% (± 7) and 797 ms (± 358) in the ASD group. No significant group differences were found on accuracy (*t*(35) = 1.1, *p* = 0.29, *d* = 0.36, BF = 0.50), nor response time (*t*(35) = 0.7, *p* = 0.48, *d* = 0.24, BF = 0.39). The two-way nested ANOVA assessing the effect of group and blocks (4 and 5) on the *I*_TOE_ revealed no effect.

**Fig. 5:**
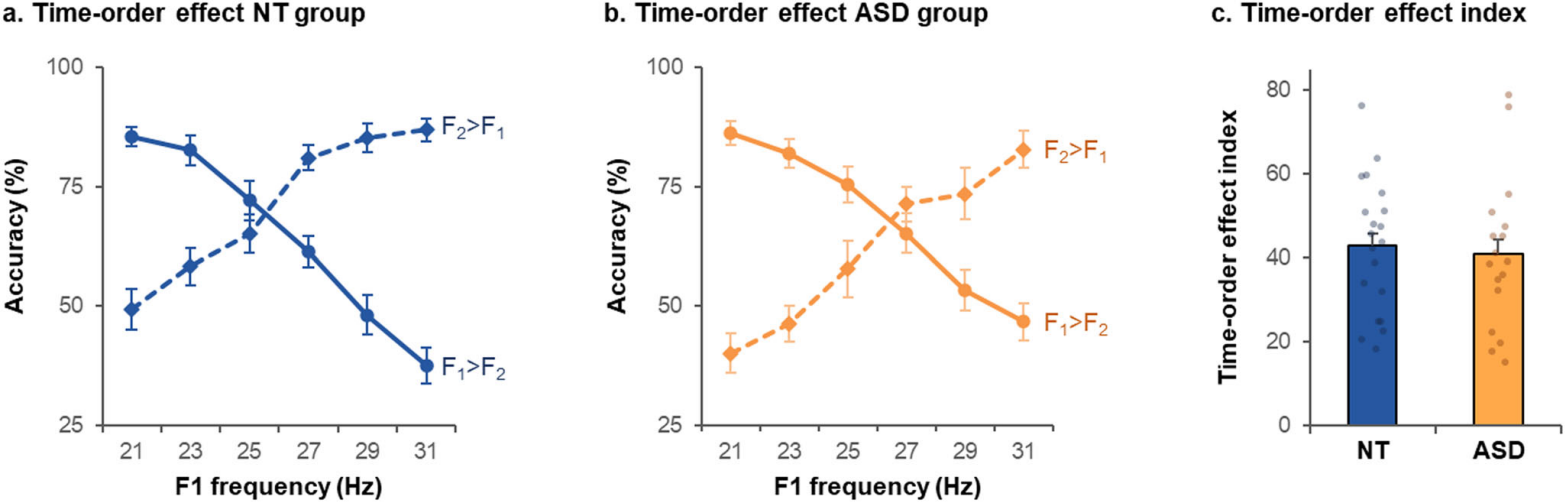
Time-order effect in Experiment III. **a**, **b** Percentage of correct answers in the TOE blocks in the neurotypical (NT) group (**a**) and autistic (ASD) group (**b**). “*F*_2_ > *F*_1_”: trials with *F*_2_ 2 Hz higher than *F*_1_, “*F*_2_ < *F*_1_”: trials with *F*_2_ 2 Hz lower than *F*_1_. **c** Time-order effect index in the TOE blocks. Error bars correspond to the standard error of the mean.

The NT and ASD groups both showed a TOE (Fig. 5a, b), with an *I*_TOE_ significantly different from zero in both NT (*I*_TOE_ = 43 ± 16, *t*(19) = 12.1, *p* < 0.0001, *d* = 2.70, BF = 4.17 × 10^7^) and ASD (*I*_TOE_ = 41 ± 18, *t*(16) = 9.3, *p* < 0.0001, *d* = 2.27, BF = 2.03 × 10^5^). The *I*_TOE_ was not significantly different between groups (*t*(35) = 0.4*, p* = 0.71, *d* = 0.12; Fig. 5c). There was anecdotal evidence in favor of no group difference (BF = 0.34). An ANOVA on *I*_TOE_ assessing the effect of frequency (low vs. high frequencies) and group revealed an interaction between group and frequency (*F*(1,35) = 5.3, *p* = 0.027), with a tendency towards higher *I*_TOE_ in lower frequencies in ASD and in higher frequencies in NT.

In Exp. III, the mean stimulus frequency of the context-setting blocks was 30 Hz, to induce a prior centered on 30 Hz, which contrasts with the mean stimulus frequency of the subsequent TOE blocks centered on 26 Hz. Investigating whether the intercept of the two accuracy curves was closer to 30 Hz (previous context) or 26 Hz (current context) may reveal a group difference in the rate at which stimulus frequency expectations were updated. In the NT group, the intercepts of the two accuracy curves were 25.4 Hz (± 1.9) in the first TOE block and 25.8 Hz (± 1.8) in the second TOE block. In the ASD group, the intercepts were 27.4 Hz (± 3.7) in the first TOE block and 26.7 Hz (± 3.8) in the second TOE block. This intercept was higher (i.e., closer to the mean stimulus frequency of the context-setting blocks) in the ASD group than in the NT group in the first TOE block (*t*(35) = 2.1, *p* = 0.045, *d* = 0.71, BF = 1.68), but not in the second TOE block (*t*(35) = 2.1, *p* = 0.045, *d* = 0.71, BF = 0.47).

In conclusion, in Exp. III, both groups implicitly learned a prior which biased their percepts, as revealed by the presence of a TOE. The extent of this perceptual bias induced by the prior did not differ between groups. Importantly, in this experiment, the mean frequency of the tactile stimulations suddenly changed (from the third to the fourth block), and both groups managed to flexibly adjust their prior expectation, but more slowly in the ASD group (i.e., it took more time for the ASD group to shift their belief from the mean frequency of context-setting blocks to the one of the TOE blocks).

### Comparison of the behavioral results in the TOE blocks in Exp. II vs. III

An ANOVA on the *I*_TOE_ with group and experiment (II vs. III) as factors revealed an interaction between group and experiment (*F*(1,66) = 5.9, *p* = 0.02), a non-significant tendency towards a group effect (*F*(1,66) = 3.0, *p* = 0.09) and no experiment effect (*p* = 0.34). As described above, the *I*_TOE_ differed between groups in Exp. II, but not in Exp. III. Between experiments, the *I*_TOE_ was larger in Exp. III than in Exp II in the NT groups (*t*(35) = 2.2, *p* = 0.018, *d* = 0.82, BF = 3.20), whereas there was no significant difference *I*_TOE_ between experiments in the ASD groups (*t*(31) = 1.0, *p* = 0.30, *d* = 0.37, BF = 0.51).

### Computational modeling approach (Exp. II and III)

The dynamic model M_1_ highlights the importance of two parameters: the sensory precision ${\pi }_{u}$, which mostly determines the average performance and controls the overall confidence in the response; and precision ratio $r={\pi }_{u}/{\pi }_{x}$ where ${\pi }_{x}$ is the precision of the prior of sameness (or sensory similarity over stimuli). The precision ratio both modulates the average performance and overall confidence, as well as it introduces a perceptual bias, which gives rise to the TOE. This bias is such that the smaller *r* (i.e., the larger ${\pi }_{x}$ with respect to ${\pi }_{u}$), the larger the TOE. Hence this model makes explicit the relative role of sensory and prior precisions.

Since sensory precision is meant to reflect the accuracy of each individual sensorium and given the fact that neither the stimulus frequency range (between 20 and 40 Hz), the stimulus intensity or duration, nor the delay between the two stimuli to be compared change over trials or experimental sessions, we assumed this parameter to be constant over the whole experiment for each subject. In contrast, the precision ratio is a context-dependent parameter and is allowed to change between sessions for a given individual.

Finally, we considered an alternative model denoted as M_0_ corresponding to the hypothesis of a very weak prior precision compared to sensory precision (or equivalently a very high sensory precision compared to prior precision)^9,10^. M_0_ is thus an extreme case of M_1_ where precision ratio $r$ tends towards infinity. It reduces to a static, unbiased, perceptual decision model. In particular, it cannot emulate a TOE (see Supplementary Fig. 2). Both M_0_ and M_1_ incorporate a response bias parameter denoted as $b$, which captures the putative individual preference for choosing an option or the other, regardless of sensory evidence.

### Bayesian Model Selection (Exp. II and III)

**Fig. 6:**
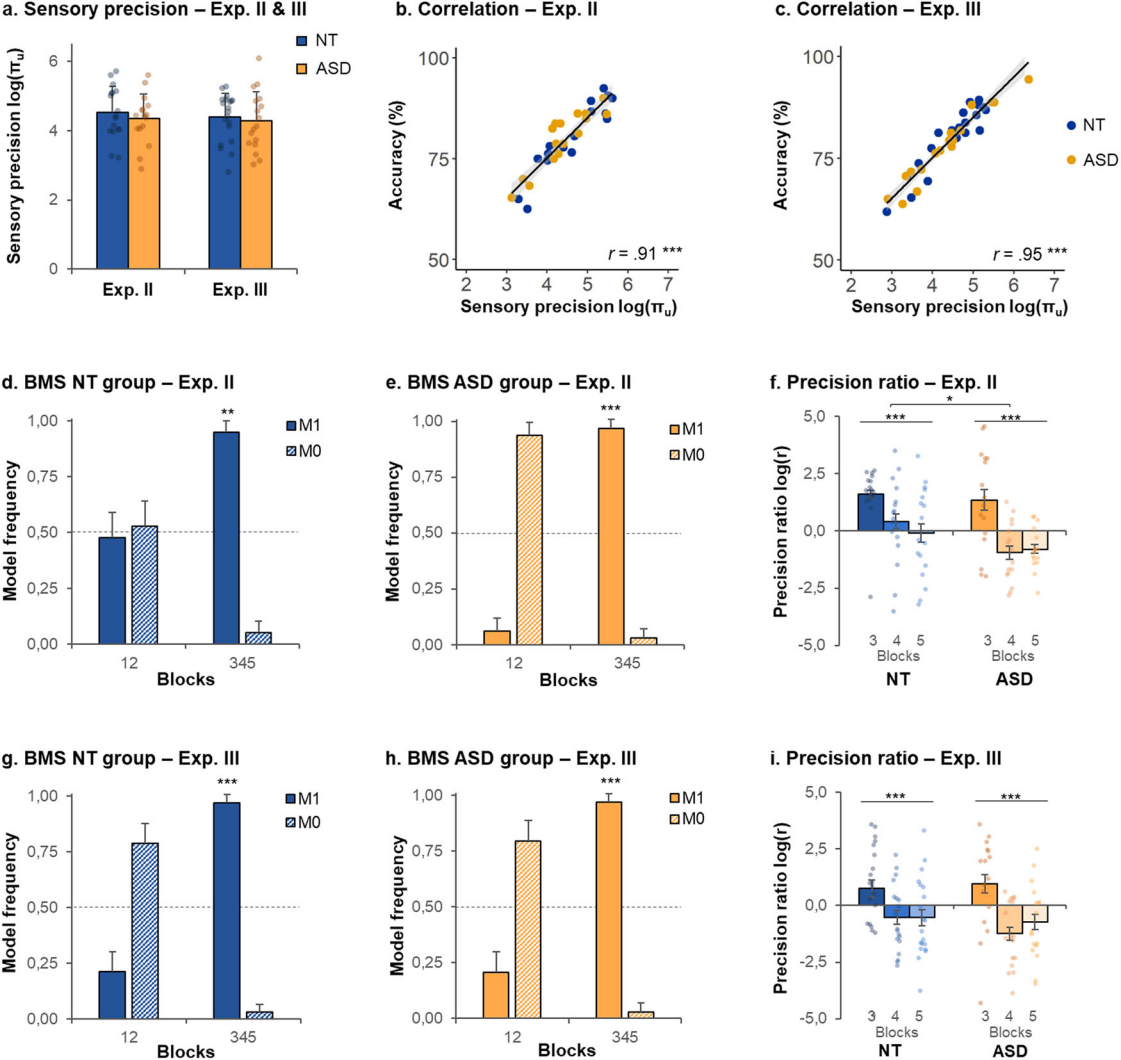
Computational results in Experiments II and III. **a** Mean sensory precision $\log ({{\rm{\pi }}}_{{\rm{u}}})$ in the two first context-setting blocks in Experiments II and III, estimated using Bayesian Model Averaging. **b**, **c** Correlations between accuracy and $\log ({{\rm{\pi }}}_{{\rm{u}}})$, both measured in the two first context-setting blocks, in Experiments II (**b**) and III (**c**). ***p < 0.0001. **d**, **e** Experiment II: Bayesian model selection (BMS) in the NT (**d**) and ASD (**e**) groups, comparing M_1_ and M_0_ in the two first context-setting blocks (12) or third context-setting and time-order effect blocks (345). Protected exceedance probability: **>0.97, ***>0.99. **f** Estimated precision ratio log(r) in Experiment II. **g**, **h** Experiment III: Bayesian model selection in the NT (**g**) and ASD (**h**) groups, comparing M_1_ and M_0_ in the two first context-setting blocks (12) or third context-setting and time-order effect blocks (345). Protected exceedance probability: ***>0.99. **i** Estimated precision ratio log(r) in Experiment III. Error bars correspond to the standard deviations, except for plots **f** and **i** where they represent the standard error of the mean. NT neurotypical, ASD autism spectrum disorders.

In Exp. II, the results of the random-effect group Bayesian Model Selection (BMS) are shown in Fig. 6d, e. In the two first context-setting blocks of Exp. II, the BMS could not identify a single best model in NT (M_0_ model frequency, exceedance probability and protected exceedance probability: 0.53, 0.59, 0.51, respectively), nor in ASD, despite a tendency toward M_0_ as the best model (model frequency, exceedance probability and protected exceedance probability: 0.94, 1.00, 0.75, respectively). In the three last blocks of Exp II, M_1_ best explained the data in the NT group (M_1_ model frequency, exceedance probability, and protected exceedance probability: 0.95, 1.00, 0.97, respectively), as well as in the ASD group (M_1_ model frequency, exceedance probability and protected exceedance probability: 0.97, 1.00, 1.00, respectively). The model comparisons did not show any group difference. Hence, the dynamic model best explained the data of both NT and ASD participants, when switching from context-setting blocks to TOE ones, i.e., when switching to a new context where the variance of the stimulus range changed while its mean value remained the same.

In Exp. III, the results of the random-effect group Bayesian Model Selection (BMS) are shown in Fig. 6g, h. In the two first context-setting blocks of Exp. III, the BMS did not clearly identify the best model in NT (M_0_ model frequency, exceedance probability and protected exceedance probability: 0.79, 1.00, 0.66, respectively) nor in ASD (M_0_ model frequency, exceedance probability, and protected exceedance probability: 0.80, 1.00, 0.69, respectively). In the three last blocks of Exp. III, M_1_ best explained the data in both the NT group (M_1_ model frequency, exceedance probability and protected exceedance probability: 0.97, 1.00, 1.00, respectively) and ASD group (M_1_ model frequency, exceedance probability and protected exceedance probability: 0.97, 1.00, 1.00, respectively). The model comparisons did not show any group difference. Here again, the dynamic model best explained the data of both NT and ASD participants when switching to a new experimental context, but this time the switch entailed a change of both the mean and variance of the stimuli.

### Parameter estimates in the two first context-setting blocks (Exp. II and III)

In Exp. II, sensory precision did not differ between groups (log$({{\rm{\pi }}}_{{\rm{u}}})$, NT: 4.5 ± 0.8; ASD: 4.3 ± 0.7; *t*(31) = 0.7, *p* = 0.46, *d* = 0.26, Fig. 6a). There was anecdotal evidence in favor of an absence of group difference in sensory precision (BF = 0.41). As expected, sensory precision was strongly correlated with the percentage of correct answers (*r* = 0.91, *p* < 0.0001, Fig. 6b). There was a group difference in response bias *b* (NT: −0.1 ± 0.2; ASD: 0.1 ± 0.2; *t*(31) = 2.8, *p* = 0.010, *d* = 0.96, BF = 5.27), that is, a tendency to answer *F*_1_ < *F*_2_ in the NT group, and *F*_1_ > *F*_2_ in the ASD group (Supplementary Fig. 3). However, note that this effect is not straightforward to interpret as the +/- response buttons were counterbalanced across participants.

In Exp. III, sensory precision did not differ between groups (log$({{\rm{\pi }}}_{{\rm{u}}})$, NT: 4.4 ± 0.7; ASD: 4.3 ± 0.9; *t*(35) = 0.5, *p* = 0.65, *d* = 0.15, Fig. 6a). There was anecdotal evidence in favor of an absence of group difference in sensory precision (BF = 0.35). Sensory precision was strongly correlated with the percentage of correct answers (*r* = 0.95, *p* < 0.0001, Fig. 6c). The response bias *b* differed between groups (NT: −0.1 ± 0.1; ASD: 0.1 ± 0.2; *t*(35) = 3.5, *p* < 0.01, *d* = 1.15, BF = 24.84) (Supplementary Fig. 3).

An ANOVA evaluating the effect of group and experiment (II or III) on sensory precision (log$({{\rm{\pi }}}_{{\rm{u}}})$), precision ratio (log(*r*)) and response bias (*b*) in the two context-setting blocks showed no effect on sensory precision nor on the precision ratio, and a group effect on the response bias (*F*(1,69) = 19.4, *p* < 0.0001) with a more negative bias in NT than ASD (i.e., a tendency to answer *F*_1_ < *F*_2_ in NT, and an opposite tendency in ASD).

Therefore, in the context-setting phase of both Experiments II and III, NT and ASD participants had similar sensory precision and precision ratio, but the two groups showed an opposite response bias.

### Parameter estimates in the last three blocks (Exp. II and III)

In Exp. II, the precision ratio was quantified and compared across groups and blocks as it was expected to change when switching context (i.e., transition from block 3 to block 4). An ANOVA assessing the effect of group and block (3, 4, 5) on the precision ratio (log(*r*)) revealed a group effect (*F*(1,98) = 5.5, *p* = 0.02), a block effect (*F*(2,98) = 13.9, *p* < 0.0001) and no significant interaction (*F*(2,98) = 1.0, *p* = 0.38). On average, in Exp. II, autistic participants had a lower precision ratio than NT (−0.14 (± 1.8) in ASD vs. 0.63 (±1.8) in NT, *t*(97) = 2.1, *p* = 0.040, *d* = 0.42, BF = 1.42, Fig. 6f), with a significant group difference in block 4 (−0.96 ± 1.3 in ASD vs. 0.40 ± 1.8 in NT; *t*(31) = 2.5, *p* = 0.020, *d* = 0.86). There was substantial evidence in favor of a lower precision ratio in ASD than NT in block 4 (BF = 3.06), and anecdotal evidence in favor of no group difference in block 5 (BF = 0.62). As compared to block 3, the precision ratio was lower in block 4 in NT (*t*(16) = 2.8, *p* = 0.014, *d* = 0.67, BF = 4.18) and in ASD (*t*(15) = 4.0, *p* < 0.005, *d* = 1.01, BF = 35.89), and was lower in block 5 in NT (*t*(16) = 3.2, *p* < 0.01, *d* = 0.77, BF = 8.47) and ASD (*t*(15) = 4.1, *p* < 0.001, *d* = 1.03, BF = 41.07). There was no difference in precision ratios between the two TOE blocks in any group (*p*-values > 0.30). As expected, the precision ratio was negatively correlated with the *I*_TOE_ in blocks 4 (*r* = −0.73, *p* < 0.0001) and 5 (*r* = −0.54, *p* = 0.001). In other words, when starting the new context (block 4) with a subtle change in experimental design in Exp. III (i.e., same stimulus mean but different variance), the ASD group expressed a higher precision ratio (between prior and sensory precisions) compared to the NT group, thus giving more weights to prior beliefs despite the implicit change in the stimulus range.

In Exp. III, an ANOVA on the precision ratio estimating the effect of group and block (3, 4, 5) showed a block effect (*F*(2,98) = 13.9, *p* < 0.0001, Fig. 6i), but no group effect or interaction (*p*-values > 0.45). Indeed, there was anecdotal evidence in favor of no group difference in precision ratio in block 4 (BF = 0.77) and block 5 (BF = 0.33). As compared to block 3, the precision ratio was lower in block 4 in NT (*t*(19) = 2.4, *p* = 0.025, *d* = 0.55, BF = 2.45) and ASD (*t*(16) = 3.8, *p* = 0.001, *d* = 0.95, BF = 22.6), and in block 5 in NT (*t*(19) = 2.3, *p* = 0.036, *d* = 0.50, BF = 1.78) and ASD (*t*(16) = 3.4, *p* < 0.005, *d* = 0.85, BF = 12.1). There was no difference in precision ratio between the two time-order effect blocks in any group (*p*-values > 0.25). As expected, the precision ratio was again negatively correlated with the *I*_TOE_ in blocks 4 (*r* = −0.55, *p* < 0.001) and 5 (*r* = −0.64, *p* < 0.0001). In sum, when there is a relatively noticeable change in the experimental context in Exp. III (e.g., a shift in the range of stimulus values), both groups adjusted their precision ratio, and no group difference was found.

We conducted an ANOVA assessing the effect of group (NT or ASD), block (3, 4 or 5) and experiment (II or III) and their interactions on the precision ratio log(*r)*. There was a group effect (*F*(1,209) = 4.7, *p* = 0.03), with a lower precision ratio in the ASD groups than in the NT groups: −0.26 (± 0.23) in ASD vs. 0.23 (± 1.9) in NT. There was a block effect (*F*(2,209) = 23.4, *p* < 0.001) with higher precision ratios in the context-setting block 3 (1.1 ± 1.9) than in the TOE blocks 4 (−0.6 ± 1.6) and 5 (−0.5 ± 1.6). There was an experiment effect (*F*(1,209) = 4.1, *p* = 0.04), with lower precision ratios in Exp. III than in Exp. II (−0.2 ± 1.9 vs. 0.26 ± 1.7). Finally, no interaction proved significant. To summarize, the sensory/prior precision ratio was modulated in both groups when changing of experimental context. It was, on average, lower in Exp. III (i.e., after a change in both stimulus mean and variance) than in Exp. II (i.e., after a change in stimulus variance only) and was lower in the ASD group (i.e., with a higher prior precision relative to sensory precision).

### Post-experiment questionnaires

The percentages of participants who reported that the first stimulus *F*_1_ was always the same were 17% (Exp. II) and 5% (Exp. III) in the NT group, and 31% (Exp. II) and 35% (Exp. III) in the ASD group, respectively. A few autistic participants proved able to explicitly identify that it was during the three first blocks that *F*_1_ was never changing (19% of ASD participants in Exp. II, and 24% of ASD participants in Exp. III), while no NT participants were able to do so. Contrary to NT, most autistic participants reported the impression that the intensity of the stimulation was stable over the blocks (67% on average in ASD vs. 35% in NT). In conclusion, ASD participants seemed to have had a more accurate perception and awareness of the experimental manipulations than NT participants, in terms of changes in stimulus frequency and intensity.

## Discussion

Predictive coding theories suggest that autistic behavior is linked to an atypical consideration and adjustment of the confidence associated with sensory information and previous beliefs, respectively^6,8–13^. These arguments are rooted in the first psychological theories of ASD^63^, but now motivate attempts to specify the underlying mechanisms using recent computational approaches to perceptual inference and learning. The ensuing mechanistic hypothesis has been summarized by George Musser as follows: “To decide what is novel, the brain needs to have in place a prior expectation that is violated. It must also assign some level of confidence to that expectation, because in a noisy world, not all violations are equal: sometimes things happen for a reason, and sometimes they just happen. The best guess scientists have for how the brain does this is that it goes through a process of meta-learning - of figuring out what to learn and what not to. According to this theory, biases in the meta-learning process explain the core features of autism. The theory essentially reframes autism as a perceptual condition, not a primarily social one; it casts autism’s hallmark traits, from social problems to a fondness for routine, as the result of differences in how the mind processes sensory input”^64^. The gist of this theoretical argument is that it underlines not only the importance of the concept of trust given to information to allow a good perception, but also the importance of the mechanisms that govern the estimation of this trust, which are based on the ability to learn and adapt to the sensory context. However, most recent theoretical descriptions, as well as the empirical studies aiming at testing them, have concentrated on the (static) imbalance between sensory precision and precision of prior belief (except for a couple of recent studies that we discuss below).

In the current study, we specifically targeted the implicit dynamics of sensory adaptation and perceptual learning, that is, adjusting the precision afforded to current beliefs and new information, respectively. To do this, we combined three behavioral experiments and compared computational models of perceptual learning and decision-making in neurotypical and autistic adults.

We used tactile discrimination tasks designed to elicit sensory adaptation and a time-order effect to characterize sensory perception in ASD and to shed light on the predictive coding hypotheses of ASD. This experimental approach was motivated by the fact that these tasks are very sensitive to key parameters, such as the sensory precision and the sensory-to-prior precision ratio. It is quite remarkable to note that the results from the three experiments contribute to drawing up a coherent picture of subtle but robust differences between autistic and neurotypical participants. We summarize and contextualize them below, first stressing the absence of difference on first-order measures (average performance), and then highlighting the importance of finer, second-order measures, and of the modeling approach to highlight and characterize the actual differences.

Reproducibly over experiments, the two groups did not differ in their absolute detection threshold, overall tactile discrimination accuracy, and reaction times. Yet, note that there was only anecdotal evidence of no group difference in accuracy and reaction times. In agreement with those direct and global observations, there was also anecdotal evidence suggesting that the two groups did not differ in the estimated sensory precision in the two frequency discrimination experiments, nor in *d’*. Former studies also reported intact tactile detection thresholds ASD^65–68^, but findings about tactile processing in ASD are quite inconsistent (see ref. ^69^ for a review). Given the absence of group difference in sensory precision, the atypical self-reported sensory sensitivity described in ASD (e.g., feeling of sensory overwhelming) may be due to unpredictable sensations, rather than due to a more acute sensory system per se^70,71^.

A strong time-order effect was observed in Exp. II and III in both neurotypical and autistic adults (extreme evidence of a time-order effect in both groups). This effect reflects a perceptual bias due to an attraction towards the mean magnitude of the stimuli. Our dynamic learning model could generate a time-order effect, contrary to the static model which considered a much larger sensory than prior precision. Importantly, this effect was not hard coded in the models, but simply emerged from viewing perception as Bayesian inference. Model comparison confirmed the ability of autistic participants to build up prior beliefs from past sensory inputs: the data of both groups and both experiments were best explained by model M_1_. There was no group difference in the model comparison, showing that both ASD and NT individuals can incorporate and use a prior.

At this stage, all these findings that are common to autistic and NT participants suffice to rule out simplistic hypotheses stating that autistic individuals constantly present with either very high sensory precision^9^ or very loose priors^10^. In other words, autistic individuals can implicitly learn the statistical regularities of their environment to build up a prior and have a typical sensory precision, suggesting that the Bayesian hypotheses of ASD should be refined.

In addition to the time-order effect blocks, the stable and long-lasting context-setting blocks used in Exp. II and III may have contributed to learning the statistics (i.e., mean and variance) of the tactile stimuli. This was observable through eliciting a perceptual bias toward the mean in both experiments. In Exp. II, the time-order effect was even found to be significantly stronger in ASD than NT. Notably, all but two autistic participants had a perceptual bias larger than the median time-order effect observed in NT participants. The consistency of this finding is quite remarkable when a large variability is usually found in empirical studies on ASD.

This interpretation is in line with the posterior model parameters showing a lower sensory/prior ratio in ASD than NT in Exp. II, in particular at the beginning of the time-order effect blocks (substantial evidence of a group difference in the first time-order effect block). A decreased ratio means a relatively higher prior precision in ASD. We hypothesize that autistic participants strongly incorporated the contextual prior induced during the preceding context-setting blocks, leading to this tight prior. This hypothesis is also consistent with autistic behaviors tending to expect very little variations between their strong expectations and their observations. Note that the reduced response time of autistic participants in the time-order effect blocks of Exp. II also speaks in favor of a higher confidence in the perceptual decisions of the ASD group, under a strong prior influence (yet note that there was only anecdotal difference in favor of a group difference in response time). Furthermore, the self-reports indicated that only some autistic participants (but no NT) noticed the subtle contextual change. Having tight priors might have contributed to their detection of the small change in Exp. II. Autistic participants might detect a state change more precisely, but without necessarily updating their prior more quickly. The NT group may have given a lesser importance to this contextual change in Exp. II, as only the variance of the stimuli was modified, which might explain the differential adjustment of the precision ratios between groups. As suggested by Van de Cruys and colleagues, “rather than having uniformly weak priors, people with autism often develop very strong priors, or expectations, in particular contexts”^72^. In the very stable context of Exp. II (i.e., no variance of the first stimulus), autistic individuals might have learnt precise priors (hyper-priors) unlikely to be applicable or generalized to other contexts. Having very tight priors can, for instance, explain categorization difficulties if low variability is allowed between items and prototype^73–75^, but also reduced perceptual flexibility in ASD^76,77^.

Interestingly though, in Exp. III, the time-order effect was not greater in the NT group than in the ASD group (anecdotal difference in favor of no group difference). In this experiment, contrary to Exp. II, the range of frequencies used in the time-order effect blocks was shifted downwards to be centered around 26 Hz instead of 30 Hz. In Exp. III, the time-order effect of the ASD group tended to be centered around higher frequencies (i.e., towards the mean frequency of the context-setting blocks) at the beginning of the time-order effect blocks. This could be, again, interpreted as a stronger effect of the priors acquired during the context-setting blocks occurring just before the time-order effect ones. However, even though this shift in time-order effect had a medium to large effect size, there was only anecdotal evidence of a group difference.

In addition, the fact that some autistic participants (but no NT) consciously perceived the change in experimental design between the context-setting and time-order effect blocks can also be interpreted as a manifestation of tight priors, which yield a higher sensitivity to subtle changes.

Although very consistent altogether, these results may look surprising as they suggest that autistic adults were even more influenced by the context than NT. An over-specific learning of the environmental structure (e.g., frequency distribution) could explain the very tight priors observed in the ASD group in Exp. II. Indeed, as the first frequency remained the same over three long consecutive blocks made of 255 trials in total, autistic individuals may have learnt a prior distribution with a very narrow variance. Likewise, some evidence in favor of an over-specific learning was found in autistic adults, associated with a lack of generalization and an increased inflexibility in ASD^37^.

Importantly here, all these findings could be understood by going beyond the analysis of averaged performance, in the light of the computational modeling of the sequence of single trial responses.

Our findings clearly establish that autistic participants do learn about the sensory context and are able to implicitly learn a prior. However, a closer look revealed that this perceptual learning is atypical, even though the group differences were quite subtle. It yielded a significantly reduced sensory adaptation in ASD than in NT participants in Exp. I (substantial evidence of a group difference), as well as evidence in favor of a reduced precision ratio in Exp. II (substantial evidence of a group difference), which also reflects a stronger expectation of sameness and slower adaptation. In Exp. I, NT participants were rapidly influenced by the context, as they showed sensory adaptation. In contrast, autistic participants may not have had enough time to build up influential priors in this relatively short time lapse. Alternatively, these results could be due to differences in neural adaptation, as a recent study showed decreased habituation to auditory or visual stimuli in autistic children^78^. Noteworthily, in Exp. I, the strong group difference in sensory adaptation was revealed despite the small number of measurement values per participant (as this procedure was initially meant to simply set a non-painful, yet perceptible, stimulus intensity for each participant).

Slower learning in ASD has also been found in studies investigating categorization. Indeed, a difference of dynamic was found in a study where autistic participants learnt prototypal categories slower than NT^79^, as items slightly differed from the prior. Furthermore, categorical learning was also found to be slower in ASD than NT^80^. It should be noted that these studies relied on paradigms where participants were trained to explicitly learn the stimulus categories, whereas the present study investigated the implicit learning of the stimulus distribution. Another study showed slower perceptual learning in autistic adults when the context changed, due to an over-specific learning of the previous context^37^. However, this study^37^ was very different from ours, as it relied on a visual search task, with an explicit change of context, and their main observations pertain to reaction times. In the current study, we further propose a mechanistic explanation through computational modeling, pointing to the central role of the precision ratio. It would be interesting to investigate whether those same mechanisms would also explain the above behavioral effect.

In Experiments II and III, we clearly observed that autistic participants did modify their perceptual process across block types, in a qualitatively similar fashion as neurotypicals. During context-setting blocks, participants in both groups did not rely much on prior information. Precision ratio was high, which is consistent with the fact that in most trials, sensory evidence was fairly large (i.e., the difference between the two frequencies was greater than in the TOE blocks). In contrast, in the TOE blocks, both groups relied much more on prior information, and precision ratio was therefore low.

Note that in Exp. II, autistic participants tended to even show a higher contrast between block types, than NT. Indeed, during context-setting blocks and contrary to what is observed in NT, static model M_0_ does capture ASD behavior significantly better than M_1_. Moreover, in subsequent TOE blocks and over both Exp. II and III, autistic participants showed a lower precision ratio than NT. This inter-session difference is to be contrasted though, with intra-session findings, namely that a lower precision ratio in the first TOE block reflects a stronger prior for sameness and a slower adjustment to this new context. Interestingly, this difference between ASD and NT participants in their precision ratios was stronger in Exp. II. Concomitantly, the anecdotal evidence showing that autistic participants were faster on average than NT is consistent with the predicted modulation of confidence by a lower precision ratio (the inverse-temperature is increased, hence the stochasticity of the decision process is decreased). Along the same lines, in Exp. III, the tendency in ASD (but not in NT) to be biased towards the mean stimulus frequency used in previous context-setting blocks could be a sign of a slower adjustment to the new frequency range.

In the ASD group, the decreased adaptation in Exp. I, stronger prior for sameness in Exp. II, and slower adjustment to the new context in Exp. III are consistent with the idea of a slower prior learning in ASD, in line with a recent study^38^. Furthermore, learning or unlearning priors more slowly would result in having more inflexible priors, consistently with the HIPPEA hypothesis^6^ and the increased inflexibility observed in autistic individuals. A difference in learning dynamics could also reconcile the differences of findings about structural vs. contextual priors in the literature about ASD^33,81^, as structural priors (i.e, pre-existing priors) are often intact in ASD, whereas contextual priors (i.e., learned priors) sometimes differ^33^. Indeed, contextual priors (i.e., learned over the time scale of an experiment) could differ between NT and ASD individuals if autistic individuals would not have had enough time to acquire or update their priors. In contrast, if prior learning is possible in ASD, but simply slower and therefore more inflexible, no group differences would be found when enough time would be given for the individuals to learn a prior, which is the case for structural priors.

A few recent studies went beyond the (static) hypotheses of atypical perceptual inference in ASD to empirically test perceptual learning. In a study investigating the perceptual bias induced by contextual framing, autistic individuals weighted recent auditory stimuli less heavily than NT individuals to form priors^38^. In contrast, they relied more on long-term statistics. In other words, autistic participants were found to slowly update their internal representation based on recent history^38^. Not only does our study replicate this general finding in a different sensory modality, but also, our experimental design combined with our modeling approach could further identify the more inflexible dynamics of perceptual learning that may subsume this observed behavior in ASD. This finding echoes the one of another study that also employed an explicit generative model of perceptual learning in ASD in an associative learning task involving visual stimuli^36^. Using hierarchical Gaussian filters to model perception under uncertainty^22^, the authors concluded from behavioral responses and changes in pupil diameters that autistic adults tend to overestimate the environmental volatility compared to NT. In line with our findings, this was associated with a slower updating of the belief about the stimulus association probability. In other words, autistic participants behaved as if uncertainty and ambiguity in the outside world were mostly attributable to expected but unpredictable changes, with the effect of down weighting the influence of any incoming prediction error. Finally, a seemingly contradictory mismatch negativity (MMN) study^82^ found that an initial model learned over the first blocks of stimulations influenced MMN amplitudes in later blocks in NT, but not in autistic adults. This absence of primacy bias in the ASD group was interpreted as evidence for faster model updating during early sensory processing in ASD^82^. To explain their findings contradicting previous work, for instance by Lieder and colleagues^38^, the authors suggested that autistic individuals might have “overcompensated for faster model updating during early sensory processing, by being more conservative on higher levels”^82^. Alternatively, to reconcile their findings with the present study and earlier studies or theories (e.g., refs. ^6,38^), we can hypothesize that their experimental context may have been too short and volatile to allow autistic participants to build up a strong enough prior, and therefore to show a primacy bias. These seemingly contradictory findings point to the complexity and subtlety of the core mechanisms behind ASD. They call for a unified theory bridging the computational, algorithmic and neuronal levels, to be tested empirically in future studies^83^. Recently, Noel and Angelaki^83^ proposed a first step towards such a unified theory. They suggested that autistic individuals would be particularly inflexible in inferring the causal structure of the environment, rather than being unable to combine likelihood and priors^83,84^. Precisely, they point to a heightened *p-common* in ASD (i.e., common prior, the tendency to combine sensory cues) that would favor simple structures/explanations over more complex ones. This is somewhat reminiscent of the strength of the prior of sameness over time in ASD, found in the current study. Moreover, we also observed that autistic participants were able to combine likelihood and priors, but in a different way, as characterized computationally by a lower precision ratio. A challenge for a unified theory will be to explain both findings.

Our study encompasses several limitations. In Exp. I, a more precise estimate of each subject’s detection threshold could possibly be obtained by using an adaptive staircase procedure. However, the use of a simple method of limits instead, enabled us to be more sensitive to an adaptation effect, since this method delivers successive stimuli that are contiguous in intensity. This result is based on only a few trials and future studies should replicate this finding using more trials. Furthermore, it should be noted that NT and ASD participants may have relied on different confidence decision criteria to give their answers in Exp. I, which might have affected the results.

In the time-order effect experiments (Exp. II and III) and due to the difficulty to recruit more ASD participants, the sample size was relatively limited. Note, however, that the time-order effect is a robust effect, including at the individual level, which explains why several previous studies using this effect could rely on even smaller groups of participants^56,85^. Nevertheless, those findings would deserve replication by future studies that would consider more participants and possibly participants who cover a larger span of the autistic spectrum. Several results showed anecdotal evidence in favor of a group difference or no group difference, which suggests that these results should be replicated with larger groups of participants or more data points.

Even though this study is based on simple sensory detection and discrimination tasks, intelligence quotient (IQ) may play a role in perceptual learning and contribute to group differences. Not having access to the IQ of our NT participants prevented us from ensuring that our two groups were fully matched in that respect. However, we matched our two groups in terms of education level (which is known to be correlated with IQ^86^, and proved to be true in our ASD sample), and found no relationship between this educational level and the observed behavioral effects, whatever the group of participants.

Finally, future studies should compare the extent of the time-order effect when tasks include or not context-setting blocks in order to precisely assess the influence of these blocks on the learning of prior knowledge.

In conclusion, a more inflexible or slower learning, which expressed in different ways over the three experiments (i.e., in ASD, decreased sensory adaptation in Exp. I, tighter prior in Exp. II, slower adjustment to the new range in Exp. III, and lack of difference in time-order effect between Exp. II and III) might be a hallmark of autistic behavior. Our findings, added to the ones obtained in different experimental contexts and sensory modalities, strengthen the idea that a slower prior learning could be a hallmark of autistic behavior. This idea is also in line with an account^87^ suggesting that ASD may be characterized by an atypical learning of predictions. Importantly, several of our results also showed that the NT and ASD groups did not differ, as autistic individuals could learn a prior and had a typical sensory precision, which does not support the apparently too simplistic hypotheses of hypo priors^10^ or higher sensory precision^9^ in ASD. Instead, we showed that prior learning was possible, globally intact, and dynamic in ASD. Yet, we evidenced some subtle specificities in favor of a slower learning and stronger priors on sameness in ASD, suggesting that the predictive coding hypotheses of ASD should be refined. Future studies combining empirical observations and hierarchical generative models of cognition in ASD will be essential to test fine hypotheses about autism^8^ and how they relate to both non-social and social symptoms of ASD^88^.

## Methods

### Participants

All participants were adults aged between 18 and 60 years. NT and ASD participants were matched in age, gender ratio, education level and handedness ratio (assessed using the Edinburgh handedness inventory). Autistic participants were diagnosed by an experienced psychiatrist specialized in autism diagnosis and in charge of the regional Resources Center for autism. They received a diagnosis of ASD according to the DSM V^1^ and scored above the cut-off threshold at the ADOS (Autism Diagnosis Observation Schedule^89^). Autistic participants presented with no language onset delay or intellectual disability (i.e., total IQ > 70, assessed using the WAIS-IV, see Table 1). All participants reported no history of neurological disorders, and none of the NT participants had a history of psychiatric disorder. Every participant completed the Autism-Spectrum Quotient (AQ) questionnaire^90^ and the Glasgow Sensory Questionnaire (GSQ)^91,92^. All participants provided informed consent prior to inclusion in the study. Approval was obtained from the local ethics committee (Southeast IV Committee for the Protection of Persons).

**Table 1:** Demographic characteristics of the participants.

	Experiment I			Experiment II			Experiment III		
	NT	ASD	*p* value	NT	ASD	*p* value	NT	ASD	*p* value
Number of participants	35	28	–	17	16	–	20	17	–
Male/Female number	27/8	20/8	ns	12/5	12/4	ns	14/6	12/5	ns
Age (years)	37 ± 10 [23–58]	33 ± 9 [18–48]	ns	30 ± 9 [18–45]	33 ± 9 [18–47]	ns	35 ± 13 [19–58]	34 ± 10 [19–48]	ns
Number of years of education after A-level	5 ± 3 [0–8]	4 ± 3 [0–8]	ns	4 ± 2 [0–8]	4 ± 3 [0–8]	ns	3 ± 2 [0–8]	4 ± 2 [0–8]	ns
AQ	12 ± 5 [4–29]	34 ± 9 [14–49]	^***^	12 ± 5 [4–21]	37 ± 8 [18–49]	^***^	12 ± 4 [6–19]	34 ± 9 [18–48]	^***^
GSQ hypersensitivity (tactile subscore)	21 ± 9 [6–50] (3 ± 1)	40 ± 16 [15–74] (6 ± 3)	^***^	23 ± 8 [15–44] (4 ± 2)	42 ± 17 [15–74] (7 ± 3)	^***^	22 ± 8 [6–38] (4 ± 1)	39 ± 17 [15–74] (6 ± 2)	^***^
GSQ hyposensitivity (tactile subscore)	17 ± 7 [5–34] (3 ± 2)	34 ± 12 [16–70] (5 ± 2)	^***^	20 ± 8 [9–37] (4 ± 2)	35 ± 14 [16–70] (6 ± 3)	^**^	19 ± 7 [6–34] (7 ± 2)	33 ± 13 [16–70] (7 ± 5)	^**^
WAIS-IV verbal comprehension score	-	125 ± 17 [83–149]	–	-	127 ± 17 [96–149]	–	-	128 ± 13 [102–149]	–
WAIS-IV perceptual reasoning score	-	111 ± 17 [78–138]	–	-	117 ± 15 [84–138]	–	-	115 ± 14 [84–138]	–

The table presents the group means ± standard deviations [range] of the participants included in the analyses.

*AQ* Autism-spectrum Quotient, *GSQ* Glasgow Sensory Questionnaire: total hypersensitivity and hyposensitivity scores, and tactile hyper/hyposensitivity subscores, *WAIS-IV* scores are missing for one participant in Exp. II and two participants in Exp. III, but their diagnosis reports mention an intelligence quotient in the normal range, *p* *p*-values correspond to the results of the Student *t*-tests and proportion tests performed between groups (ns: non-significant if *p* > 0.05, *p < 0.05, **p < 0.01, ***p < 0.001).

There were three experiments relying on tactile detection or discrimination tasks with NT and ASD adults. Experiment I measured tactile detection thresholds. Experiments II and III relied on a tactile 2AFC task designed to elicit a time-order effect, with a stable stimulus frequency range in Experiment II, and a changing stimulus frequency range in Experiment III. Here follows the description of the ASD and NT groups of subjects who participated in each experiment. Some participants were involved in the three experiments (2 NT and 12 ASD), but a period of at least 10 months separated their participation to Experiments II and III.

In Exp. I (tactile threshold measurement), there were thirty-five NT participants and 28 autistic participants. The two groups were matched for age, education level, and sex ratio (Table 1).

In Exp. II (TOE with a stable stimulus range), there were eighteen NT and 20 autistic participants. Participants who got less than 56.5% of correct answers (confidence interval, according to binomial law for *p* = 0.05 and 120 successes out of 240 trials) during the context-setting blocks were excluded from the analyses, which resulted in the exclusion of one NT participant and four autistic participants. Among the excluded participants, one NT and one ASD participant reported being hyposensitive due to damaged fingers, and three ASD participants reported being hyperreactive to the tactile stimulations. After exclusion, it resulted in groups of 17 NT participants and 16 autistic participants (Table 1).

In Exp. III, there were twenty NT and 20 autistic participants. Three autistic participants were discarded from the analyses as they got <56.5% of correct answers during the context-setting blocks. Among the excluded ASD participants, one was hyperreactive to the tactile stimulations, one reported a high fatigability, and one was disturbed by the sensation produced by the adhesive tape. After exclusion of these three participants, it resulted in a group of 20 NT adults and a group of 17 autistic adults (Table 1).

### Tactile stimuli

Two gold electrodes were placed on the first and third phalanx of the left index finger of the participant. Electrical stimulations were delivered by a constant current stimulator (GRASS technology). The electrical stimulation was felt on the internal face of the third phalanx (cathode electrode). Stimulations were composed of 5 ms-long boxcar pulses, repeated at a given frequency. The overall tactile stimulation lasted 500 ms. Tactile stimuli were generated using Matlab 2013b.

### Experiment I: tactile detection task

Each experiment was programmed using the software package Presentation (Version 17.1, Neurobehavioral Systems, www.neurobs.com). The tactile detection threshold was estimated in each participant, prior to Exp. II and III. The aim was twofold: (i) matching the subjective stimulation intensity between participants for the subsequent discrimination task; (ii) comparing NT and ASD participants in terms of both the mean and the variance of this threshold. The stimulus frequency was constant and set to 30 Hz. The intensity of the electrical stimulation was gradually increased and then decreased (method of limits, Fig. 1a). Each participant was asked to report when the tactile sensation started to be perceived (detection, during the increase of stimulus intensity) as well as when the sensation disappeared (disappearance, during the decrease). Starting from 0 mA, the stimulus intensity was increased by 0.02 mA every second. Participants were told to raise their non-stimulated hand, as soon as they could feel the stimulation and this first detection threshold value was collected. We kept on increasing the intensity until reaching 1.5 times this threshold value. Then, we used the same progression to decrease the intensity and participants had to lower their non-stimulated hand as soon as the sensation would disappear. This procedure was repeated twice, resulting in four measures of threshold: two of detection and two of disappearance. Data were collected specifically for Exp. I, or prior to Exp. II or III. If one participant participated in both Exp. II and III, then only the first measure was kept (i.e., from Exp. II).

The threshold measurement procedure involved a prolonged exposure to tactile stimulations, and might thus be significantly influenced by sensory adaptation^93^. If so, detection of a change should be biased differently, depending on whether the stimulus intensity is being increased or decreased. Precisely, detection of the stimulation should occur at a lower intensity than the feeling of its disappearance. We assessed the sensory adaptation effect by computing, in each participant, the averaged difference between the disappearance and detection intensities. We tested whether the sensory adaptation effect was significantly different from zero in each group and compared these effects between groups.

### Experiments II and III: tactile discrimination tasks

The intensity of the electrical stimulations aimed to be at 2.5 times their detection threshold^94^. Yet, for some NT participants (7 in Exp. II, 14 in Exp. III) and autistic participants (15 in Exp. II, 15 in Exp. III), we allowed a slight modulation of this factor to guarantee a comfortable (non-painful and non-itchy) but clearly perceived stimulus. This ended up with similar multiplying factors between the two groups: 2.4 in the NT group and 2.2 in the ASD group on average (no significant difference), and mean intensities of 1.3 mA (±0.4) in the NT group and 1.1 mA (±0.4) in the ASD group (no significant difference). The intensity was kept constant during the whole task.

At each trial, participants received two successive stimulations to be compared in frequency (Fig. 1b). Each stimulation lasted for 500 ms and the time interval between the two stimulations was fixed and equal to 2000 ms. A white fixation cross was displayed on the center of the computer screen during stimulations and delay. Right after the delivery of the second stimulation, a question mark appeared on screen to indicate that an answer was expected. Participants had up to 4 seconds to answer by clicking on the computer mouse buttons, using their dominant hand. If the second stimulation was perceived as higher in frequency than the first one, they had to click on the side of the plus sign (+), as shown on screen. Conversely, if it was perceived as lower, they had to click on the side of the minus sign (−). Finally, a black screen appeared after the mouse click and the next trial would start after a fixed inter-trial interval of 1500 ms. The sides corresponding to the plus (+) and minus (–) answers respectively, were indicated on screen at each trial and never changed for a given participant but were counterbalanced over participants. No feedback was provided.

Participants were told to prioritize accuracy over speed. Each experiment consisted of 447 trials, and each trial lasted for 5.3 sec on average. It was divided into five blocks (7 to 8 minutes each): three context-setting blocks, followed by two time-order effect blocks. Participants began the task after having completed a 16-trial long training made of one sample of each possible pair of stimuli that would be presented during the context-setting blocks.

### Experiments II and III: design of the context-setting blocks

The initial blocks (1, 2, 3) aimed at familiarizing the participants with the task and at inducing a context-based prior on the expected range of stimulus frequencies. During these first three blocks, the frequency of the first stimulation (*F*_1_) was always the same and equal to 30 Hz. The frequency of the second stimulation (*F*_2_) was 0 to 8 Hz higher or lower than *F*_1_ (*F*_2_ = 22 to 38 Hz). The 17 possible pairs of frequencies were repeated five times each per block. Each block thus consisted of 85 trials: 40 trials in which *F*_2_ was higher than *F*_1_, 40 trials where *F*_2_ was lower than *F*_1_ and 5 trials where *F*_2_ was equal to *F*_1_ (the latter were discarded from the analyses). By keeping the first stimulation constant, we induced a strong prior expectation of 30 Hz stimulation for *F*_1_. The order of the trials was pseudo-randomized. Precisely, pairs of stimuli were presented, one cycle after another. A cycle corresponding to the set of all possible pairs of stimuli. Within each cycle, the trial order was fully randomized.

### Experiments II and III: design of the TOE blocks

Blocks 4 and 5 aimed at eliciting a time-order effect (TOE). Participants were not informed that the design of these two blocks was different from the one in the three preceding blocks. Each block consisted of 96 trials with eight presentations of each of the 12 new pairs of frequencies. Trials were pseudo-randomly ordered (successive trials were never identical). Importantly, to induce a TOE and regardless of the experiment, the frequency *F*_1_ was no more constant but, within a trial, the absolute difference in frequency between the two stimuli was always equal to 2 Hz.

In Exp. II, the frequency of the first stimulation could take six different values with equal probability (*F*_1_ = 25, 27, 29, 31, 33, or 35 Hz; a range centered at 30 Hz). The second stimulation was then 2 Hz higher or lower than *F*_1_, with equal probability (*F*_2_ = 23, 25, 27, 29, 31, 33, 35, or 37 Hz).

In Exp. III, the frequency of the first stimulation could take another six values with equal probability (*F*_1_ = 21, 23, 25, 27, 29, or 31 Hz; a range centered at 26 Hz). The second stimulation was then 2 Hz higher or lower than *F*_1_, with equal probability (*F*_2_ = 19, 21, 23, 25, 27, 29, 31, or 33 Hz).

In order to quantify the TOE at the individual level, we define a TOE index denoted as I_TOE._ Over all possible *F*_1_ frequencies, it is simply given by the average difference in accuracy between the case where *F*_2_ was 2 Hz higher than *F*_1_ and the case where it was 2 Hz lower (e.g., 25 Hz – 27 Hz vs. 25 Hz – 23 Hz), finally normalized by the individual mean accuracy over all trials. Note that this index has no unit and is equal to zero in the absence of TOE. The greater the TOE is, the greater the *I*_TOE_ is. To further describe the TOE, we also calculated the intercept of the two accuracy curves (*F*_2_ > *F*_1_ and *F*_2_ < *F*_1_) in the first and second halves (i.e., in blocks 4 and 5).

### Computational modeling approach

We developed a probabilistic model of Bayesian perception and decision making in the context of 2AFC. This model emphasizes the role of sensory and *prior* precisions in the way past sensory information is incorporated into prior beliefs and influences the current perceptual decision. It turns out that this simple and general hypothesis of a Bayesian learning process is sufficient to elicit a time-order effect. We here describe in detail the perceptual and decision parts of this model, respectively, as well as our model fitting procedure.

### Perceptual model

At each trial $t$, the subject is asked to compare two stimulus frequencies presented sequentially and denoted by ${u}_{1}^{(t)}$ and ${u}_{2}^{(t)}$, respectively. To perform this perceptual decision, the subject has to infer the actual feature of interest for each stimulus, based on noisy sensory inputs, as well as on acquired prior beliefs. An optimal fashion to combine these two information is given by Bayes rule, which simply weights each information by its afforded confidence (precision or inverse variance). In several experimental contexts, perceptual decisions have been well captured by a Bayesian inference process^56^. However, what is often overlooked is how these confidence weights are themselves estimated and adjusted over trials, in a context-dependent fashion. For instance, in a 2AFC, if sensory inputs are noisy and if the two stimuli to be compared are quite similar, prior information should play a significant role, as suggested by the observed time-order effect behavioral accuracy. To capture this process, one needs to appeal to Bayesian learning, a between-trial mechanism by which posterior estimates resulting from Bayesian inference at a given trial, become the prior belief at the next trial for subsequent inference. The proposed perceptual model implements Bayesian learning in the context of a sequence of two stimulus-feature comparisons and makes explicit the key role of two parameters: (i) sensory precision ${\pi }_{u}$; and (ii) precision ratio $r$ which controls the relative contribution of sensory inputs and prior expectations on perception (see below).

We denote by ${x}_{n}$ the feature to be inferred from the *n*^*th*^ stimulus of the sequence (be it the first or second stimulus of a given trial). It relates to the sensory input through the likelihood function:

1 $${u}_{n} \sim N\left({x}_{n},{\pi }_{u}^{-1}\right)$$

Besides, subjects entertain a prior belief over ${x}_{n}$ which is built on past observations. It writes and can be decomposed as follows:

2 $$p\left({x}_{n}|{U}_{1\ldots n-1}\right)=\int p\left({x}_{n}|{x}_{n-1}\right).p\left({x}_{n-1}|{U}_{1\ldots n-1}\right)d{x}_{n-1}$$

where ${U}_{1\ldots n-1}$ indicates all sensory inputs presented before stimulus *n*.

The above decomposition highlights two terms. The first one can be referred to as a prior of sameness. It quantifies how much the subject believes that consecutive stimuli resemble each other. The second one is the posterior belief over the previous stimulus feature, given the whole sequence of stimuli so far. Without loss of generality, we consider Gaussian distributions for both:

3 $$\left\{\begin{array}{c}p\left({x}_{n},|,{x}_{n-1}\right)=N\left({x}_{n-1},{\pi }_{x}^{-1}\right)\\ p\left({x}_{n-1},|,{U}_{1\ldots n-1}\right)=N\left({\mu }_{n-1},{\pi }_{{\mu }_{n-1}}^{-1}\right)\end{array}\right.$$

Equation (3) introduces three model variables. ${\pi }_{x}$ is the prior precision, such that the higher the prior precision, the stronger the belief of sameness. ${\mu }_{n-1}$ and ${\pi }_{{\mu }_{n-1}}$ are state variables in the sense that they evolve over stimulus presentations. They designate the mean and precision of the posterior belief over the (n-1)^th^ stimulus, respectively. The prior of sameness term and the information inherited from past observations combine according to Eq. (2), to form the full prior which is also Gaussian, and whose first moment (${\theta }_{(n)}$) and second moment (${\pi }_{{\theta }_{(n)}}$) write:

4 $$\left\{\begin{array}{c}{\theta }_{(n)}={\mu }_{n-1}\\ {\pi }_{{\theta }_{(n)}}={\left({\pi }_{{\mu }_{n-1}}^{-1}+{\pi }_{x}^{-1}\right)}^{-1}=\frac{{\pi }_{{\mu }_{n-1}}.{\pi }_{x}}{{\pi }_{{\mu }_{n-1}}+{\pi }_{x}}\end{array}\right.$$

Note that the prior of sameness moderates the Bayesian learning process, in the sense the weaker the prior of sameness, the weaker the influence of the current posterior belief on subsequent inference. In other words, the strength of the prior not only depends on the confidence over the current belief, but is further modulated by the overall belief in contextual stability.

Finally, having defined the likelihood (Eq. (1)) and prior (Eq. (4)), applying Bayes rule yields the following formula for the two moments of the Gaussian posterior distribution over ${x}_{n}$:

5 $$\left\{\begin{array}{c}{\pi }_{{\mu }_{n}}={\pi }_{u}+{\pi }_{{\theta }_{n}}={\pi }_{u}+\frac{{\pi }_{{\mu }_{n-1}}.{\pi }_{x}}{{\pi }_{{\mu }_{n-1}}+{\pi }_{x}}\\ {\mu }_{n}=\frac{{\pi }_{u}.{u}_{n}+{\pi }_{{\theta }_{n}}.{\mu }_{n-1}}{{\pi }_{u}+{\pi }_{{\theta }_{n}}}\end{array}\right.$$

Note that here, contrary to Eq. (4) where variances were adding up, sensory and prior precisions add up.

Interestingly, Eq. (5) can be rewritten to emphasize the learning process, as follows:

6 $$\left\{\begin{array}{c}{\mu }_{n}={\mu }_{n-1}+{\tau }_{n}.\left({u}_{n}-{\mu }_{n-1}\right)\\ {\tau }_{n}=\frac{{\pi }_{u}}{{\pi }_{{\mu }_{n}}}=\frac{{\pi }_{u}}{{\pi }_{u}+{\pi }_{{\theta }_{n}}}\end{array}\right.$$

where precision ratio ${\tau }_{n}$ plays the role of a learning rate. It takes values between 0 and 1, and the closer to one, the larger the update of the subject’s belief based on the latest sensory input. The latter happens when prior precision ${\pi }_{{\theta }_{n}}$ is neglectable compared to sensory precision. Conversely, if prior precision is much greater than ${\pi }_{u}$, then ${\tau }_{n}$ gets close to zero and the belief is not updated.

To fully grasp how learning rate ${\tau }_{n}$ evolves over trials, let us rewrite the bottom line of Eq. (6) as follows:

7 $${\tau }_{n}=\frac{1}{1+\frac{{\pi }_{{\mu }_{n-1}}.{\pi }_{x}}{{\pi }_{u}.\left({\pi }_{{\mu }_{n-1}}+{\pi }_{x}\right)}}=1-\frac{1}{1+{\tau }_{n-1}+r}$$

with precision ratio $r=\frac{{\pi }_{u}}{{\pi }_{x}}$.

A non-trivial consequence here is that the dynamic Bayesian learning process is convergent and fully determined by the value of the parameter $r$. Indeed, Eq. (7) defines a recurrent series for the learning rate, which converges towards a limit value ${\tau }_{\mathrm{lim}}$ given by:

8 $${\tau }_{\mathrm{lim}}=\frac{-r+\sqrt{r.\left(r+4\right)}}{2}$$

Note that the higher $r$, the closer to one ${\tau }_{\mathrm{lim}}$. Moreover, it can be shown that the speed of convergence towards ${\tau }_{\mathrm{lim}}$ also increases with $r$. This means that the smaller the prior precision with respect to sensory precision, the faster the adjustment of the learning rate and the larger the limit value hence the weight afforded sensory input for perceptual inference.

### Response model

The subject’s response at trial *t* relies on the inferred probability that the first stimulus has a higher frequency than the second one. This posterior probability writes:

9 $$p\left({x}_{t}^{(1)} > {x}_{t}^{(2)},|,{U}_{1\ldots t}\right)=p\left({x}_{t}^{(1)}-{x}_{t}^{(2)} > 0,|,{U}_{1\ldots t}\right)=1-{F}_{\left({x}_{t}^{(1)}-{x}_{t}^{(2)},|,{U}_{1\ldots t}\right)}$$

where ${U}_{1\ldots t}$ indicates all past stimulations including those presented in current trial *t*, and *F* stands for the cumulative distribution function.

Given the above perceptual model, the posterior distribution over the difference ${x}_{t}^{(1)}-{x}_{t}^{(2)}$ is Gaussian and the above probability then writes:

10 $$p\left({x}_{t}^{(1)} > {x}_{t}^{(2)},|,{U}_{1\ldots t}\right)=\frac{1}{2}.\left(1+{erf}\left({R}_{t}\right)\right)$$

where *erf* indicates the error function and its argument ${R}_{t}$ is given by:

11 $${R}_{t}={\alpha }_{t}.\left({\mu }_{t}^{(1)}-{\mu }_{t}^{(2)}\right)$$

with:

12 $${\alpha }_{t}=\sqrt{\frac{{\pi }_{{\mu }_{t}}^{(1)}.{\pi }_{{\mu }_{t}}^{(2)}}{2.\left({\pi }_{{\mu }_{t}}^{(1)}+{\pi }_{{\mu }_{t}}^{(2)}\right)}}$$

where ${\mu }_{t}^{(1)}$ (resp. ${\mu }_{t}^{(2)}$) and ${\pi }_{{\mu }_{t}}^{(1)}$ (resp. ${\pi }_{{\mu }_{t}}^{(2)}$) are the posterior mean and precision pertaining to the first (resp. second) stimulus in trial *t*. Finally, given the above perceptual model and Eq. (7) in particular, one can rewrite the two terms of the right-hand side in Eq. (10) as follows:

13 $$\left({\mu }_{t}^{(1)}-{\mu }_{t}^{(2)}\right)=\left({u}_{t}^{(1)}-{u}_{t}^{(2)}\right)-\left(1-{\tau }_{t}^{\left(1\right)}.{\tau }_{t}^{\left(2\right)}\right).\left({u}_{t}^{\left(1\right)}-{\mu }_{t}^{\left(0\right)}\right)+\left(1-{\tau }_{t}^{\left(1\right)}\right).\left({u}_{t}^{\left(2\right)}-{\mu }_{t}^{\left(0\right)}\right)$$

and:

14 $${\alpha }_{t}=\sqrt{\frac{{\pi }_{u}}{2.\left({\tau }_{t}^{\left(1\right)}+{\tau }_{t}^{\left(2\right)}\right)}}$$

where ${\mu }_{t}^{\left(0\right)}$ is the prior mean at the beginning of trial *t* (i.e., prior to observing first stimulus ${u}_{t}^{(1)}$). ${\tau }_{t}^{\left(1\right)}$ and ${\tau }_{t}^{\left(2\right)}$ are the learning rates that pertain to each stimulus in trial *t*, respectively.

Interestingly, Eqs. (10) and (11) show that the decision variable ${R}_{t}$ is homogeneous to a *t*-statistics or a *d*-prime measure, i.e., to the ratio of a mean contrast and its associated standard deviation. Furthermore, the higher a positive (resp. negative) ${R}_{t}$, the higher (resp. the lower) the probability of answering that ${u}_{t}^{(1)}$ is greater than ${u}_{t}^{(2)}$. Precisely, obtaining a high probability in favor of either one answer or the other rests on both having a high contrast (a high difference ${\mu }_{t}^{(1)}-{\mu }_{t}^{(2)}$) and a high confidence weighting ${\alpha }_{t}$. Importantly though, the contrast term relates to the veridical physical difference between the two stimuli (${u}_{t}^{(1)}-{u}_{t}^{(2)}$), but also depends on two additional terms that depend on the two learning rates and act as perceptual biases. Similarly, the confidence weighting term depends on sensory precision ${\pi }_{u}$, but is also modulated by the two learning rates. Note that coefficient ${\alpha }_{t}$ is also similar to an inverse-temperature coefficient, as typically used in probabilistic decision models^95^.

Finally, the response model predicts the subject’s binary decision at trial *t* as the outcome of a *Bernoulli* process with probability ${q}_{t}$ of answering that ${u}_{t}^{(1)}$ is greater than ${u}_{t}^{(2)}$ given by:

15 $${q}_{t}=\frac{1}{2}.\left(1+{erf}\left({R}_{t}+b\right)\right)$$

where additional parameter $b$ captures a putative response bias.

Hence, the proposed full model rests on two perceptual parameters (sensory precision ${\pi }_{u}$ and precision ratio *r*) and one response parameter (response bias *b*). In addition, the initial values for the two state variables, ${\mu }_{0}^{\left(0\right)}$ and ${\tau }_{0}^{\left(0\right)}$ have to be defined, but it turns out that beyond the few first trials, they have no influence whatsoever on the overall subject’s performance, which fully depends upon the above three parameters.

In this study, we compare this full Bayesian learning model (denoted M_1_) with a static model (denoted M_0_), where the perceptual decision is purely based on the veridical contrast (${u}_{t}^{(1)}-{u}_{t}^{(2)}$) and sensory precision ${\pi }_{u}$ such that:

16 $${R}_{t}=\frac{\sqrt{{\pi }_{u}}}{2}.\left({u}_{t}^{(1)}-{u}_{t}^{(2)}\right)$$

Note that M_0_ is what would be observed if *r* would tend towards infinity in the above full model, that is when the influence of priors is infinitely weak (i.e., the precision of the prior for sameness tends towards zero). M_0_ is a static model with no state variables and only two parameters (sensory precision ${\pi }_{u}$ and response bias *b*).

While M_0_ cannot produce a TOE and is thus not expected to well explain subject’s responses in blocks 4 and 5, it may well explain subject’s responses in another experimental context, namely during the context-setting blocks 1, 2, and 3. In the latter, in most trials, the difference between the two stimuli to be compared is fairly large and prior information should not contribute much to the perceptual decisions.

### Model fitting

To implement, fit and compare models between experimental conditions and groups, as well as estimating relevant parameters using Bayesian Model Averaging (BMA), we used the VBA Matlab toolbox (http://mbb-team.github.io/VBA-toolbox/)^96^. We also assessed the reliability of our modeling approach in performing simulation-based confusion and model identifiability analyses (see Supplementary Methods 1).

Data were fitted on the two first context-setting blocks to estimate the sensory precision and on the three last blocks to capture the transition between the third context-setting block and the two TOE blocks.

When fitting the models on the behavioral data, we excluded the trials whose RTs were outliers (i.e., more than three standard deviations away from the participant’s mean). In the first two blocks and in the last three blocks, the percentages of excluded trials were 1.2% (± 1.6) and 0.8% (± 0.7) in NT and 0.8% (± 0.9) and 0.7% (± 1.2) in ASD in Exp. II, and 0.4% (± 0.5) and 0.6% (± 0.7) in NT, and 0.4% (± 0.6) and 0.7% (± 0.8) in ASD in Exp. II, respectively. The R-squared values associated with the fits are given as Supplementary Table 1.

The sensory precision was estimated from the two first context-setting blocks, an estimate that was then used as a prior for the multisession inference in the three last blocks. The supplementary information contains the considered priors over model parameters (Supplementary Table 2), the model posterior parameters (Supplementary Table 3) and the probabilities of answer for each trial type and model (Supplementary Fig. 2).

### Questionnaires

In addition to the AQ and GSQ questionnaires, participants answered a task-related questionnaire at completion of the study (Exp. II or III), to report about their first-person experience of the task. They were asked to rate the overall difficulty (globally and block-wise). They were also asked to specify: (i) whether they had the feeling that the intensity of the stimuli varied over time; (ii) whether the first or second stimulation of each trial remained constant in frequency (if so, in which block), and (iii) whether they thought they had answered “plus” or “minus” most frequently. Finally, they were free to add any comment about the strategies they might have used to perform the task.

### Statistical analyses

Descriptive statistics are reported as mean ± standard deviation. Demographic data and tactile detection thresholds were compared between groups using unpaired t-tests. Psychometric functions were estimated in the context-setting blocks using a general linear model with a logit link function. Differential thresholds were determined as the difference in frequency between *F*_1_ and *F*_2_ to reach 75% of correct answers. Within-group repeated measure ANOVAs were used to assess the accuracy and response times over the five blocks, in Exp. II and III, and Tukey’s t-tests were used post-hoc. TOE blocks were also analyzed with two-way nested ANOVAs, with the factor participant nested in the factor group and two main factors: group and TOE blocks. To estimate the intercept of the TOE, we first assessed the performance bias for each frequency *F*_1_, by computing the difference in response accuracy between *F*_2_ > *F*_1_ trials and *F*_2_ < *F*_1_ trials. Then, the intercept of the two accuracy curves in TOE blocks was assessed by fitting robust linear regressions to this performance bias as a function of *F*_1_ frequency, participant by participant. The mean intercept was calculated as the average of participants’ intercepts, after excluding the few outliers (i.e., intercepts estimated outside the range of presented frequencies: 3 NT and 1 ASD in Exp. II, and 1 NT and 1 ASD in Exp. III). Correlations between the questionnaire scores and inferred model parameters were assessed using Pearson’s *r*. A Pearson’s *r* of 0.10 is considered as a small effect, 0.30 as a medium effect, and 0.50 as a large effect. Effect sizes are reported as Cohen’s *d*: very small (*d* = 0.01), small (*d* = 0.20), medium (*d* = 0.50), large (*d* = 0.80), or very large (*d* > 1.20) effect sizes^97,98^. Additionally, we computed Bayes factors (BF) for the main effects of interest using the BayesFactor package in R (with default settings). To interpret the magnitude of this effect, we refer to Jeffreys (1961)^99^ and Kass & Raftery (1995)^100^ classifications. Unanswered trials were excluded from all the above analyses (they represented less than 0.01% of all recorded trials). All statistical analyses were performed using R (version 2.15.3, http://www.r-project.org/). The threshold for statistical significance was set to *p* < 0.05.

### Reporting summary

Further information on research design is available in the Nature Research Reporting Summary linked to this article.

### Supplementary information

Supplementary information

Reporting summary

## Data Availability

The data generated in this study is deposited in the Zenodo database: 10.5281/zenodo.10075516.

## Code availability

The codes used in this study are available from the corresponding author, L.A.S.T., upon reasonable request.
