# Supplementary material for: Disentangling sensory precision and prior expectation of change in autism during tactile discrimination
Source: NPJ Sci Learn. 2023 Dec 6;8:54. doi: 10.1038/s41539-023-00207-5 (PMC10700558; doi:10.1038/s41539-023-00207-5)
Supplement: Supplementary file 1 — Supplementary information [file 41539_2023_207_MOESM1_ESM.pdf]

Supplementary information

Supplementary Methods 1

Model validation. This section provides a thorough evaluation of the reliability of our modelling approach. This entails evaluating both the discriminability of alternative perceptual decision-making models and the identifiability of model parameters [1]. Therefore, we used realistic Monte Carlo simulations, and we then followed the same analysis steps as with our empirical data, namely:

(i) Context Setting (CS) data analysis (2 blocks)

a. Random-effect Bayesian model comparison of M_0 and M_1, having simulated the data under each model alternatively to compute a confusion matrix.

b. Estimating the sensory precision and response bias for each synthetic individual using Bayesian model averaging (BMA) to assess the identifiability of these two parameters.

(ii) Time-order effect (TOE) data analysis (2 blocks)

a. Random-effect Bayesian model comparison of M_0 and M_1, having simulated the data under each model alternatively to compute a confusion matrix. Here, individual sensory precisions take approximately the same value as in the two previous Context Setting blocks and are assumed to be known thanks to the previous analysis.

b. Estimating the precision ratio for each subject and each block to assess the identifiability of this parameter.

c. Estimating again the response bias to assess reproducibility of this estimation.

Data simulations

To simulate data, we considered model M_0 and model M_1 as the true model, successively, and for each we generated 50 synthetic experiments of 15 subjects each. For each subject, using the same design as in Exp. II, we generated data of two Context Setting blocks and two TOE blocks. A single value for π_u and for b were drawn for each subject, independently, while one value per session was drawn for precision ratio r (see table below for the parameters of the generative gaussian distributions).

[1] Wilson, R. C. & Collins, A. G. Ten simple rules for the computational modeling of behavioral data. *eLife* **8**, e49547 (2019).

Model parameter	Mean	Variance
$log(\pi_u)$	4.6	0.5
$log(r)$<br>(M_1 only)	1 (CS sessions)<br>-1 (TOE sessions)	0.5<br>0.5
b	0	0.1

Mean and variance values of the Gaussian distributions to generate the parameter values for each synthetic subject's dataset.

Model discriminability

Figures below show the confusion matrices obtained from fitting the simulated data with models M_0 and M_1, successively, on the CS and TOE data, respectively.

For CS blocks, it appears clearly that when data are generated with model M_0 the identified most probable model over the 50 synthetic experiments is always the true model M_0. Conversely, when model M_1 is used to generate the data, M_0 and M_1 are estimated as the most likely model with equal probability. This speaks to the poor discriminability power of the CS design to distinguish between M_0 and M_1, especially when precision ratio is relatively high as we observed empirically for these block types. This further emphasizes the fact that when most trials show a fairly large difference between stimuli, priors have a negligible influence onto the perceptual decision. This also justifies the Bayesian model averaging strategy to estimate the sensory precision and response bias.

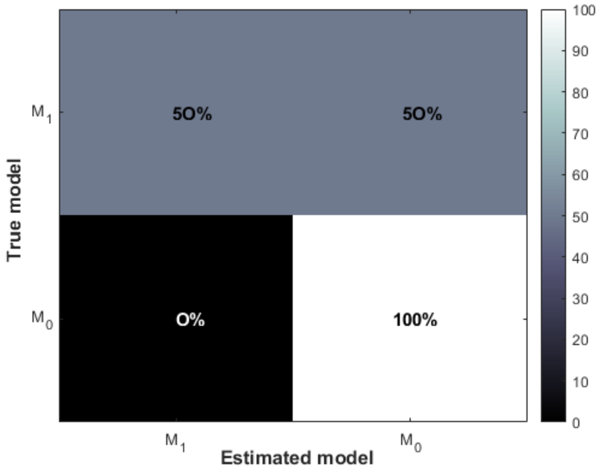

Confusion matrix obtained with a Context Setting design, after simulating under each true model 50 experiments with 15 subjects each. In each cell is indicated the percentage of experiment for which, given that M_0 (or M_1) was the true model, M_0 (or M_1) was estimated as the most likely generative model.

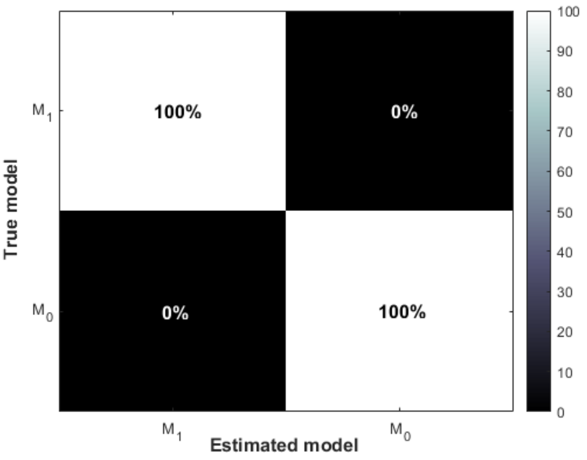

Confusion matrix obtained with a Time-Order Effect design, after simulating under each true model 50 experiments with 15 subjects each. In each cell is indicated the percentage of experiment for which, given that M_0 (or M_1) was the true model, M_0 (or M_1) was estimated as the most likely generative model.

For TOE blocks, the obtained confusion matrix shows that the true model was always identified as the most likely generative model, on all the 50 simulated experiments. This contrasts with the confusion matrix obtained with the Context Setting design. It confirms that, since M_1 is the only model that can reproduce a time-order effect, M_0 and M_1 can easily be distinguished when a design known to elicit this effect, is used.

Model identifiability

The figure below shows the estimated sensory precision values against the true simulated ones, for all the synthetic subjects and experiments (N = 50 x 15 = 750). The parameter values estimated via BMA on context setting data are very close to the true ones (all dots lie in the close vicinity of the first diagonal). The correlation between the two is 0.85.

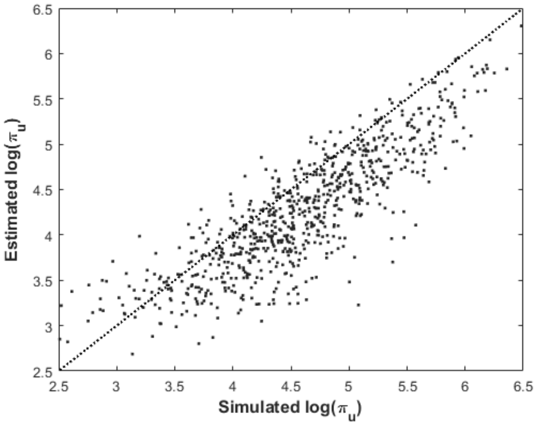

Estimated versus simulated sensory precision values for each subject of each synthetic experiment.

Similarly, the plots below shows the estimated precision ratio against the true simulated ones, but here for each block separately. The estimation is less precise than the one of the sensory precision (with a correlation around 0.2), probably partly because it is based on fewer trials. However, the estimation remains fairly accurate, both for Context Setting and TOE blocks. In particular, the difference in precision ratio between the two block types is found.

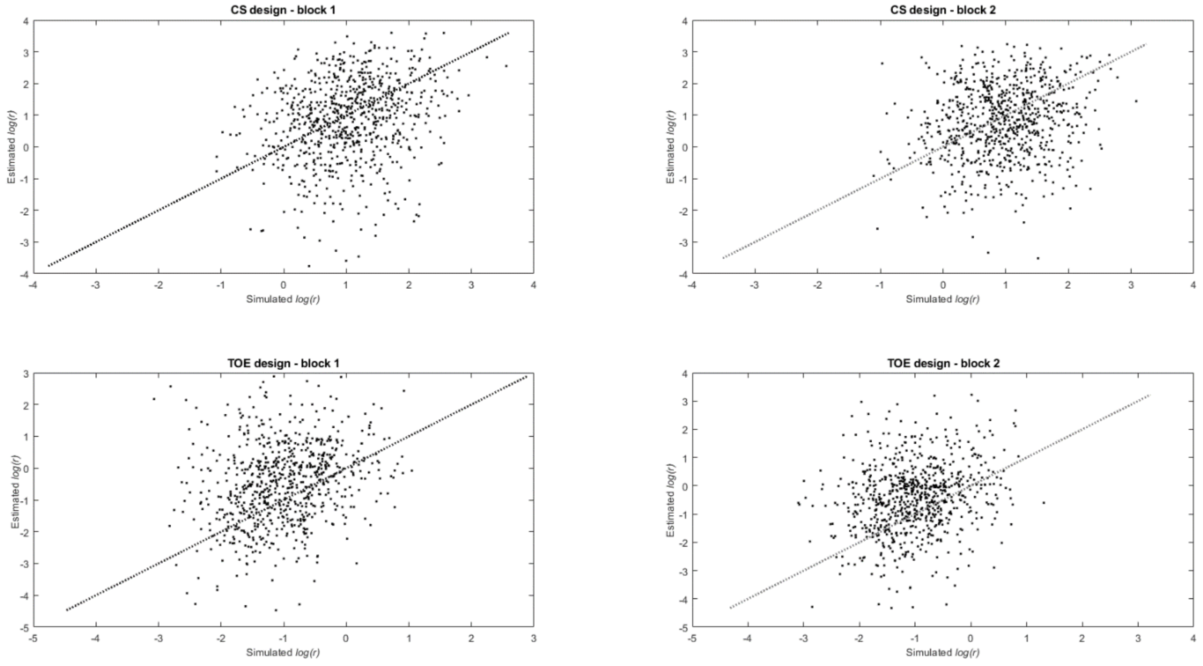

Estimated vs. simulated precision ratio for each subject of each synthetic experiment, and separately for Context Setting blocks (upper row) and TOE blocks (lower row).

Finally, the figure below shows the estimated response biases as a function of the true simulated ones, for both the Context Setting blocks (left plot), and TOE blocks (right plot). With both designs, except for the very few extreme values that tend to be underestimated, the response bias is estimated with very high accuracy (the correlation is higher than 0.98 in both cases).

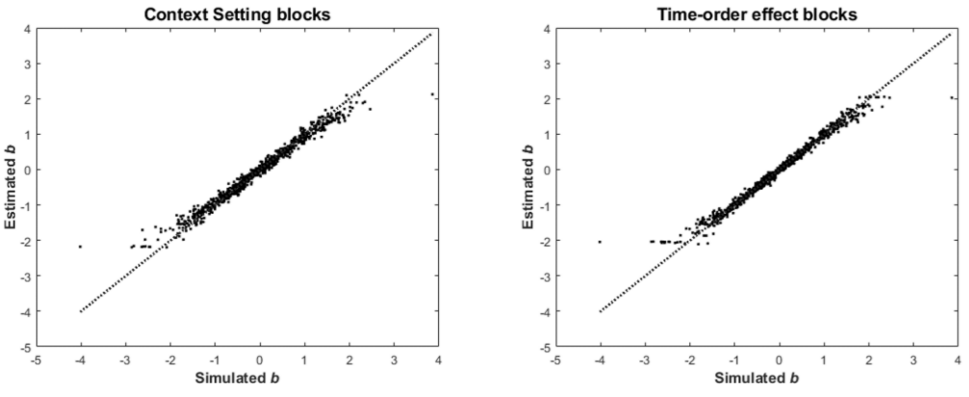

Estimated vs. simulated response bias, for Context Setting (left plot) and TOE (right plot) blocks.

Supplementary Table 1

	Experiment II				Experiment III			
	Blocks 1 2		Blocks 3 4 5		Blocks 1 2		Blocks 3 4 5	
	M_1	M_0	M_1	M_0	M_1	M_0	M_1	M_0
**NT**	0.44 (± 0.20)	0.42 (± 0.21)	0.33 (± 0.14)	0.28 (± 0.13)	0.43 (± 0.17)	0.41 (± 0.17)	0.32 (± 0.13)	0.23 (± 0.11)
**ASD**	0.40 (± 0.17)	0.39 (± 0.17)	0.29 (± 0.14)	0.20 (± 0.09)	0.38 (± 0.21)	0.37 (± 0.21)	0.28 (± 0.17)	0.20 (± 0.14)

R-squared values for each population and model (means ± standard deviation).

Supplementary Table 2

	M_1	M_0
**Observation parameter**		
$\boldsymbol{b}$	m = 0 (σ = 1)	
**Evolution parameters**		
$\boldsymbol{\pi_u}$	m and σ = inferred from the two first context-setting blocks	
$\boldsymbol{r}$	m = 0 (σ = 20)	*n.a.*
**State variables**		
$\boldsymbol{\mu_0^{(0)}}$	m = log(30) (σ = 0.05)	*n.a.*
$\boldsymbol{\pi_{\mu_0}^{(0)}}$	m = log(16) (σ = 0.1)	*n.a.*

Mean (m) and variance (σ) of the Gaussian priors over observation parameters, model parameters and initial state variables in the fit on the 3rd, 4th and 5th blocks in Experiments II and III. n.a.: not applicable.

Supplementary Table 3

**Experiment II**				
	**M_1**		**M_0**	
	**NT**	**ASD**	**NT**	**ASD**
**Observation parameter**				
b	-0.1 (± 0.2)	0.1 (± 0.2)	-0.1 (± 0.2)	0.1 (± 0.2)
**Evolution parameters**				
**$\log(\pi_u)$**	5.0 (± 0.7)	4.8 (± 0.9)	4.7 (± 0.7)	4.3 (± 0.6)
**$\log(r)$**	0.6 (± 1.3)	-0.1 (± 1.1)	*n.a.*	*n.a.*
**Experiment III**				
	**M_1**		**M_0**	
	**NT**	**ASD**	**NT**	**ASD**
**Observation parameter**				
b	-0.1 (± 0.1)	0.1 (± 0.2)	-0.1 (± 0.1)	0.0 (± 0.2)
**Evolution parameters**				
**$\log(\pi_u)$**	4.8 (± 0.7)	4.6 (± 1.0)	4.3 (± 0.7)	4.1 (± 0.9)
**$\log(r)$**	-0.1 (± 1.2)	-0.4 (± 1.2)	*n.a.*	*n.a.*

Model posteriors after the fits on the 3rd, 4th and 5th blocks in Experiments II and III. In M_1, the parameters were averaged over the three blocks. n.a.: not applicable.

Supplementary Figure 1

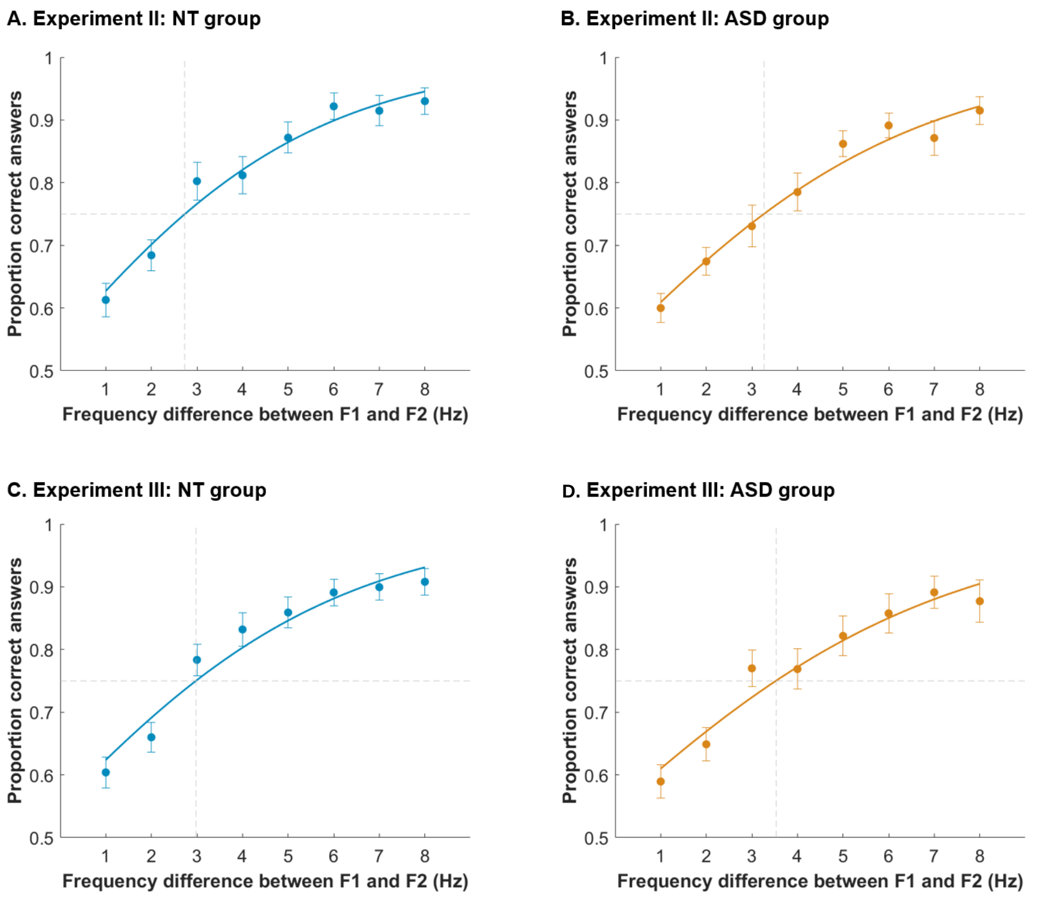

Psychometric curves in the NT (blue) and ASD (orange) groups in Exp. II (A-B) and Exp. III (C-D). Error bars indicate the standard error of the mean. The dashed grey line indicates the relative detection threshold to reach 75% of correct frequency discrimination.

Supplementary Figure 2

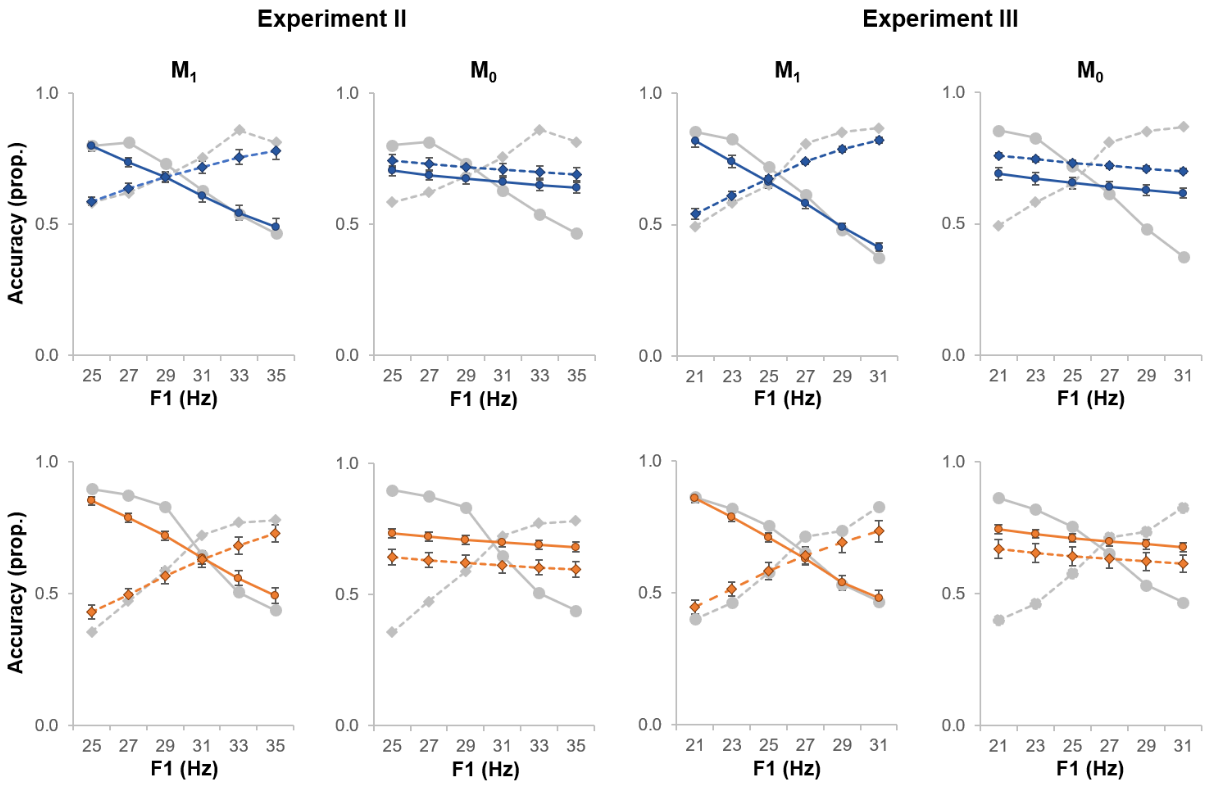

Probability of giving a correct answer for each experiment, model and group (the NT group is displayed in blue, and the ASD group in orange). In each plot, the estimation by the model is given in color (blue or orange) and the actual group data are overlapped in grey. Full line: trials with F_2<F_1, dashed line: trials with F_2>F_1. Error bars indicate the standard error of the mean.

Supplementary Figure 3

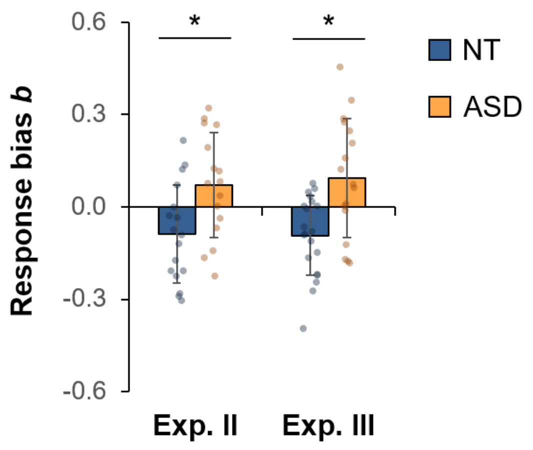

*Model parameter b, capturing the putative individual preference for choosing a certain answer, regardless of sensory evidence. A negative response bias indicates a tendency to answer F1 < F2, while a positive response bias indicates a tendency to answer more frequently F1 > F2. Data from the two first context-setting sessions in Experiment (Exp.) II and III in the NT (blue) and ASD (orange) groups. Error bars indicate standard deviations. * p < .01*
