# Supplementary material for: Disentangling sensory precision and prior expectation of change in autism during tactile discrimination
Source: NPJ Sci Learn. 2023 Dec 6;8:54. doi: 10.1038/s41539-023-00207-5 (PMC10700558; doi:10.1038/s41539-023-00207-5)
Supplement: Supplementary file 2 — Reporting summary [file 41539_2023_207_MOESM2_ESM.pdf]

nature portfolio

Corresponding author(s): Laurie-Anne Sapey-Triomphe

Last updated by author(s): Nov 6, 2023

Reporting Summary

Nature Portfolio wishes to improve the reproducibility of the work that we publish. This form provides structure for consistency and transparency in reporting. For further information on Nature Portfolio policies, see our Editorial Policies and the Editorial Policy Checklist.

Statistics

For all statistical analyses, confirm that the following items are present in the figure legend, table legend, main text, or Methods section.

n/a	Confirmed	
☐	☒	The exact sample size (n) for each experimental group/condition, given as a discrete number and unit of measurement
☐	☒	A statement on whether measurements were taken from distinct samples or whether the same sample was measured repeatedly
☐	☒	The statistical test(s) used AND whether they are one- or two-sided<br>*Only common tests should be described solely by name; describe more complex techniques in the Methods section.*
☐	☒	A description of all covariates tested
☐	☒	A description of any assumptions or corrections, such as tests of normality and adjustment for multiple comparisons
☐	☒	A full description of the statistical parameters including central tendency (e.g. means) or other basic estimates (e.g. regression coefficient) AND variation (e.g. standard deviation) or associated estimates of uncertainty (e.g. confidence intervals)
☐	☒	For null hypothesis testing, the test statistic (e.g. F, t, r) with confidence intervals, effect sizes, degrees of freedom and P value noted<br>*Give P values as exact values whenever suitable.*
☐	☒	For Bayesian analysis, information on the choice of priors and Markov chain Monte Carlo settings
☒	☐	For hierarchical and complex designs, identification of the appropriate level for tests and full reporting of outcomes
☐	☒	Estimates of effect sizes (e.g. Cohen's d, Pearson's r), indicating how they were calculated

Our web collection on statistics for biologists contains articles on many of the points above.

Software and code

Policy information about availability of computer code

Data collection	Data were collected using the software package Presentation (Version 17.1, Neurobehavioral System, www.neurobs.com)
Data analysis	Data were extracted using Matlab 2015a, and statistical analyses were performed using R (version 2.15.3, http://www.r-project.org/). Computational models were executed using custom codes in Matlab 2015a and using the VBA Matlab toolbox (http://mbb-team-github.io/VBA-toolbox/).

For manuscripts utilizing custom algorithms or software that are central to the research but not yet described in published literature, software must be made available to editors and reviewers. We strongly encourage code deposition in a community repository (e.g. GitHub). See the Nature Portfolio guidelines for submitting code & software for further information.

Data

Policy information about availability of data

All manuscripts must include a data availability statement. This statement should provide the following information, where applicable:

- Accession codes, unique identifiers, or web links for publicly available datasets
- A description of any restrictions on data availability
- For clinical datasets or third party data, please ensure that the statement adheres to our policy

The source data that support the findings of this study are deposited in the repository 10.5281/zenodo.10075516.

Human research participants

Policy information about studies involving human research participants and Sex and Gender in Research.

Reporting on sex and gender	The sex of the participants was determined based on self-reporting. In each experiment, the sex-ratio was was matched between groups. Experiment I involved 27 NT male and 8 NT female participants, as well as 20 ASD male and 8 ASD female participants. Experiment II involved 12 NT male and 5 NT female participants, as well as 12 ASD male and 4 ASD female participants. Experiment III involved 14 NT male and 6 NT female participants, as well as 12 ASD male and 5 ASD female participants. The sex-ratio of our participants was close to the sex-ratio encountered in ASD (1 female for 4 male). No sex-based analyses were performed because the sample size was too small to make comparisons between men and women, and because we did not have any prior hypotheses on an effect of sex on perceptual learning.
Population characteristics	See above.
Recruitment	Individuals with ASD who had received a diagnosis in the Regional Center for autism and who recently interacted with this center, who did not meet any exclusion criteria, and were matching the inclusion criteria were invited to participate using an invitation letter. We only recruited participants with no intellectual disability. Some of the neurotypical participants were acquaintances of the researchers, so we cannot exclude a bias of recruitment, but we tried to recruit participants from diverse backgrounds (for instance, with education levels ranging from high-school diploma level to PhD level, as in the ASD group).
Ethics oversight	Ethical approval was obtained from the local ethics committee (South East IV Committee for the Protection of Persons).

Note that full information on the approval of the study protocol must also be provided in the manuscript.

Field-specific reporting

Please select the one below that is the best fit for your research. If you are not sure, read the appropriate sections before making your selection.

☐ Life sciences ☒ Behavioural & social sciences ☐ Ecological, evolutionary & environmental sciences

For a reference copy of the document with all sections, see nature.com/documents/nr-reporting-summary-flat.pdf

Behavioural & social sciences study design

All studies must disclose on these points even when the disclosure is negative.

Study description	This study encompasses three behavioral experiments that assess tactile perception in adults with and without autism. The tactile stimulations were non-painful electrical stimulations delivered on the finger. In Experiment I, participants simply had to report the appearance or disapperance of the tactile stimulation, in order to measure a detection threshold and to assess an adaptation effect. In Experiments II and III, participants performed a two-alternative forced-choice task where they had to compare the frequencies of two successive stimulations. All data were quantitative data, except for a short debriefing questionnaires performed at the end of Experiments II and III that provided some qualitative information. We used computational modeling to further characterize the behavioral quantitative responses.
Research sample	All participants were adults aged between 18 and 60 years. Experiment I involved 35 neurotypical subjects (8 females, mean age: 37 years old) and 28 subjects with autism (8 females, mean age: 33 years old). Experiment II involved 17 neurotypical subjects (5 females, mean age: 30 years old) and 16 subjects with autism (4 females, mean age: 33 years old). Experiment III involved 20 neurotypical subjects and (6 females, mean age: 35 years old) and 17 subjects with autism (5 females, mean age: 34 years old). Participants with and without autism were matched in age, gender ratio and education level. The gender ratio was close to the ratio encoured in the autism population (about 1 girl for 4 boys). Volunteers with autism spectrum disorder had received their diagnosis from a psychiatrist specialized in autism diagnosis and in charge of the regional Resources Center for autism, and were contacted by this psychiatrist. The neurotypical volunteers have been recruited so as to match the demographic characteristics of the autism group. Participants had no intellectual disability.
Sampling strategy	The sampling procedure of the ASD participants was random, but based on the fact that it involved people with ASD who had received a diagnosis in the regional center for autism. The sampling procedure of the neurotypical participants was pseudo-random as it aimed at matching the demographic characteristics of the ASD group.
Data collection	The material involved during the data collection was two gold electrodes placed on the first and third phalanx of the left index finger of the subject using tape, and connected to a constant current stimulator (GRASS technology). Participants were sitted at about 60cm from the computer screen (Dell) with the computer mouse in the right hand to give their answer using the left or right click. Besides the participant and the experimenter no one else was present during data collection. Within one experiment, the experimental conditions were the same for all participants. The experimenter was not blind to the experimental condition or design.
Timing	Data from Experiments I and II were collected between March and June 2014. Data from Experiment III were collected between February and May 2015.

Data exclusions	In Experiment I, no participant or data were excluded. In Experiments II and III, a total of seven participants with ASD and one neurotypical participant were excluded. The criteria to define which participants would be excluded was if subjects got less than 56.5% of correct answers (confidence interval, according to binomial law for p = .05 and 120 successes out of 240 trials) during the context setting blocks, as the main interest was to study the time-order effect blocks following these context-setting blocks. The participants who were excluded reported that they were too hypersensitive to the stimuli (4 ASD participants), too hyposensitive due to damaged fingers (1 neurotypical and 1 ASD participants), too tired to focus (1 ASD) or too disturbed by the sensation produced by the adhesive tape that maintained the electrode on the finger (1 ASD participant). The exclusion criteria were pre-established.
Non-participation	No participant dropped out.
Randomization	There was no randomization. Individuals with a diagnosis of Autism Spectrum Disorder were assigned to the ASD group, and neurotypical individuals (who reported no history of neurological or psychiatric disorders) were assigned to the neurotypical group.

Reporting for specific materials, systems and methods

We require information from authors about some types of materials, experimental systems and methods used in many studies. Here, indicate whether each material, system or method listed is relevant to your study. If you are not sure if a list item applies to your research, read the appropriate section before selecting a response.

Materials & experimental systems

n/a	Involved in the study
☒	☐ Antibodies
☒	☐ Eukaryotic cell lines
☒	☐ Palaeontology and archaeology
☒	☐ Animals and other organisms
☒	☐ Clinical data
☒	☐ Dual use research of concern

Methods

n/a	Involved in the study
☒	☐ ChIP-seq
☒	☐ Flow cytometry
☒	☐ MRI-based neuroimaging
